# Spatial sexual dimorphism of X and Y homolog gene expression in the human central nervous system during early male development

**DOI:** 10.1186/s13293-015-0056-4

**Published:** 2016-01-12

**Authors:** Martin M. Johansson, Elin Lundin, Xiaoyan Qian, Mohammadreza Mirzazadeh, Jonatan Halvardson, Elisabeth Darj, Lars Feuk, Mats Nilsson, Elena Jazin

**Affiliations:** Department of Organismal Biology, EBC, Uppsala University, Uppsala, Sweden; Science for Life Laboratory, Department of Biochemistry and Biophysics, Stockholm University, Stockholm, Sweden; Department of Immunology, Genetics and Pathology, Uppsala University, Uppsala, Sweden; Department of Women’s and Children’s Health, International Maternal and Child Health, Uppsala University, Uppsala, Sweden; Department of Public Health and General Practice, Norwegian University of Science and Technology, Trondheim, Norway

**Keywords:** Sex differences, Human embryo development, Female, Male, Gene expression, Protocadherin, Neuroligin, X chromosome, Y chromosome, Brain, Spinal cord, Cortex, Medulla oblongata, Rolling circle amplification, *PCDH11X*, *PCDH11Y*, *NLGN4X*, *NLGN4Y*, *ISLET1*, *OLIG2*, *SOX10*, *NeuN*

## Abstract

**Background:**

Renewed attention has been directed to the functions of the Y chromosome in the central nervous system during early human male development, due to the recent proposed involvement in neurodevelopmental diseases. PCDH11Y and NLGN4Y are of special interest because they belong to gene families involved in cell fate determination and formation of dendrites and axon.

**Methods:**

We used RNA sequencing, immunocytochemistry and a padlock probing and rolling circle amplification strategy, to distinguish the expression of X and Y homologs in situ in the human brain for the first time. To minimize influence of androgens on the sex differences in the brain, we focused our investigation to human embryos at 8–11 weeks post-gestation.

**Results:**

We found that the X- and Y-encoded genes are expressed in specific and heterogeneous cellular sub-populations of both glial and neuronal origins. More importantly, we found differential distribution patterns of X and Y homologs in the male developing central nervous system.

**Conclusions:**

This study has visualized the spatial distribution of PCDH11X/Y and NLGN4X/Y in human developing nervous tissue. The observed spatial distribution patterns suggest the existence of an additional layer of complexity in the development of the male CNS.

**Electronic supplementary material:**

The online version of this article (doi:10.1186/s13293-015-0056-4) contains supplementary material, which is available to authorized users.

## Background

The current concept about the mechanism of sexual differentiation of the brain during development includes not only the action of gonadal hormones but also the function of genes encoded by the sex chromosomes [1–3]. The brain effects mediated by the actions of the Y chromosome have not attracted the same amount of interest as those mediated by genes encoded on the larger X chromosome, which is enriched in genes involved in neurodevelopment and cognition [4]. In recent years, attention to Y chromosome functions in the brain during development has increased, due to its proposed involvement in autism [5] and non-syndromic speech delay [6]. Furthermore, neuroimaging analysis within a rare cohort of humans with diverse sex chromosome aneuploidies showed that the X and Y chromosomes have opposing effects on cortical development [7] and cortical thickness asymmetry [8].

We have shown that several Y chromosome-encoded genes are expressed in the brain during human male development [9]. Of these, *PCDH11Y* and *NLGN4Y* deserve a special attention because they belong to two gene families named protocadherins and neuroligins, predominantly expressed in the nervous system and with important functions in survival, cell fate determination and formation and maturation of synapses in different neuronal populations [10, 11]. *PCDH11Y* is unique to humans while *PCDH11X* is present in our closest relatives, the chimpanzees. The duplication event that transferred the *PCDH11X* to the Y chromosome took place approximately six MYA ago [12]. During early investigations of *PCDH11Y,* when the gene was described as the only “gain of function” gene in the human genome, there was an ongoing debate regarding its human specificity [13]. The question was finally resolved in 2006 when it was shown that *PCHD11Y* is human-specific, and absent in both gorilla (*Gorilla gorilla*) and chimpanzee (*Pan troglodytes*) as well as other non-human primates [14].

*NLGN4Y*, together with its X homolog counterpart, has been proposed as candidate genes for autism spectrum disorders [5, 15]. The PCDH11X/Y gene pair has been suggested to be involved in psychosis, partly because of its putative roles in human speciation and partly due to its possible relation to structural changes in the brain [16, 17]. An important step to understand the possible functional relevance of Y-encoded genes for the development of sexual dimorphism in the brain is to investigate whether X and Y homologs are expressed in a spatial sex-specific manner.

Until now, no research has been done on the genetic contribution of X- and Y-linked genes to sex differences in the human brain prior to hormonal activation. It is therefore of great interest to investigate eventual sexual dimorphisms contributed by sex chromosome-linked genes alone with minimal influence of hormones [2]*.* To detect the contribution of genetic components encoded on the Y chromosome to sex differences in the brain, we used a human central nervous system (CNS) tissue acquired as early as possible during development, so that androgen hormones, such as testosterone, are not expressed or expressed at minimum levels. Indeed, in the developing Sertoli cells in the testes, androgens start to be produced around week 11 post-gestation and slowly start to influence gene expression in the brain in an activating manner after week 11 [2, 18–21].

These experiments cannot be performed using conventional *in situ* hybridization or immunofluorescence techniques because of cross-hybridization of probes due to high sequence similarity [15]. Indeed, different isoforms of *PCDH11Y* share 97 to 99 % sequence identity with the coding region of the homolog *PCDH11X* isoforms. Both *in situ* messenger RNA (mRNA) detection [22] and immunohistochemistry [23] have shown widespread expression of *PCDH11X* and *PCDH11Y* in the human brain during development, but the contribution of each gene to the observed signals could not be distinguished.

Different isoforms of *NLGN4Y* share 89 to 98 % sequence identity with the coding region of the homolog *NLGN4X* isoforms. RT-PCR experiments in individual male and female adult human brain tissues determined that both *NLGN4X* and *NLGN4Y* are expressed [24], but the spatial expression patterns of each gene, particularly during human brain development, is not known. To specifically distinguish the tissue and cellular distribution of transcripts from X and Y homolog genes, we designed a strategy using RNA sequencing technology, together with immunohistochemistry and padlock probing in combination with rolling circle amplification (RCA) [25]. As this last method relies on dual-target recognition and ligation-dependent signal amplification, it can be adapted to discriminate highly similar targets such as splice variants, mutations or members of the same gene family which may differ by only one or a few single nucleotide sequences [26]. We show here that both *PCDH11Y* and *NLGNY* are expressed in specific cellular sub-populations in the CNS that more rarely express their X homolog genes. Using a combination of immunohistochemistry and padlock probing, we also show that cells of different origin express *PCDH11Y* and *NLGNY* in the CNS during early male human development.

## Results

### RNA sequencing of medulla oblongata and midbrain samples from female and male human embryos

**Fig. 1:**
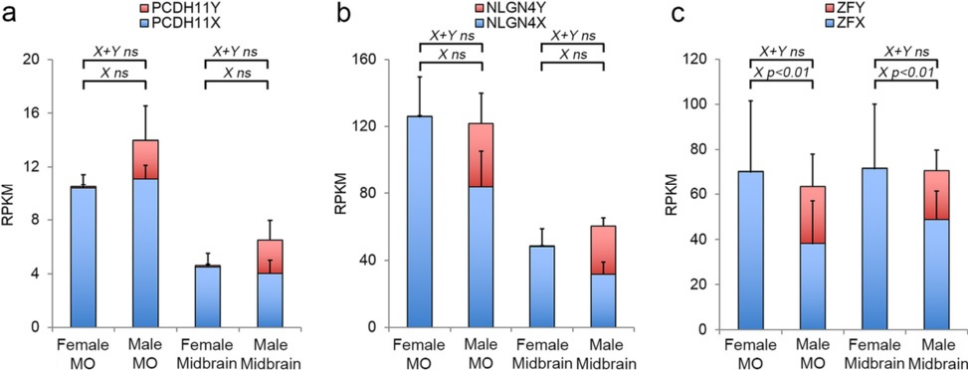
Quantification of expression levels using RNA sequencing. *Staple bars* show the average RPKM for both total RNA and polyA+ sequencing of female and male MO and midbrain for PCDH11X/Y (**a**), NLGN4X/Y (**b**) and ZFX/Y (**c**). Only ZFX showed significant expression differences between females and males in both tissues, and NLGN4X presented a non-significant increase in females

To quantify the expression of X and Y transcripts for *PCDH11* and *NLGN4* with high resolution and to study the expression of the other members of the neuroligin and protocadherin families, we performed RNA sequencing of CNS samples from female and male embryos, at 8–11 weeks of development. Both total RNA and polyA+ RNA were sequenced as described in the “Methods” section. Additional file 1: Table S1 shows the results of the analysis for all genes belonging to these gene families. All results are expressed in RPKM (reads per kilobase of transcript per million mapped reads). A very low number of RPKM from the Y-linked genes were detected in female tissue confirming the specificity of the technique (Fig. 1a, b). None of the X-linked genes showed significant differential expression levels in males and females in either the medulla oblongata (MO) or midbrain. To confirm the accuracy of the RNA sequencing data, we also compared the expression of *PCDH11X* and *NLGN4X* with the expression of *ZFX*, previously reported in adult brain as an escapee from X inactivation [27]. Figure 1c shows that *ZFX* had about twice the expression levels in females compared to males (adjusted *p* value 0.006) agreeing with previous results [27].

### Padlock probes specifically discriminate between X and Y homologs

**Fig. 2:**
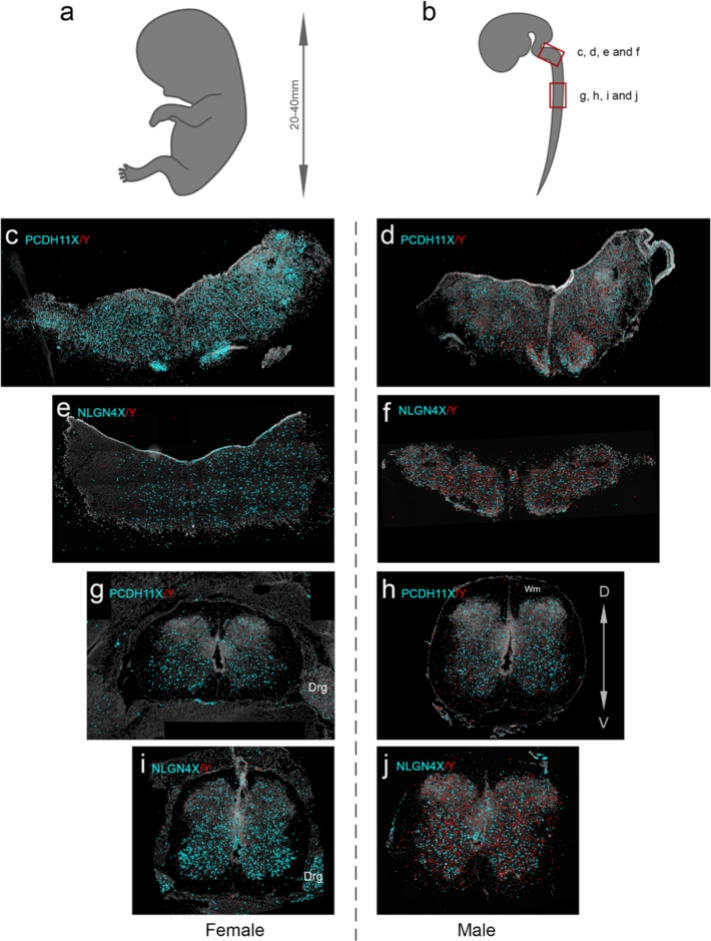
Padlock probe hybridization can distinguish between expression of X and Y homolog genes for *PCDH11* and *NLGN4*. **a**, **b** Schematic representation of an embryo modified from Gasser [47] and the CNS of an embryo at 12 weeks of gestation modified from His [48]. The *boxes in red* mark the approximate position of the coronal sections shown in the rest of the figure. **c** Female medulla oblongata (MO) section hybridized with padlock probes for PCDH11X and Y. X signals in *cyan* and Y signals in *red* for all subfigures. **d** Male MO section hybridized with padlock probes for PCDH11X and Y. **e** Female MO section hybridized with padlock probes for NLGNX and Y. **f** Male MO section hybridized with padlock probes for NLGNX and Y. **g** Female spinal cord (SC) section hybridized with padlock probes PCDH11X and Y. **h** Male SC section hybridized with padlock probes for PCDH11X and Y. **i** Female SC section hybridized with padlock probes NLGNX and Y. **j** Male SC section hybridized with padlock probes for NLGNX and Y. All signals from females and males were detected using a Zeiss Axio Imager.Z2 epi-fluorescence microscope. The images were produced using the Zen software and enhanced to 15–20-pixel dots to allow visualization in ×20 magnification pictures. *Drg* dorsal root ganglia

**Fig. 3:**
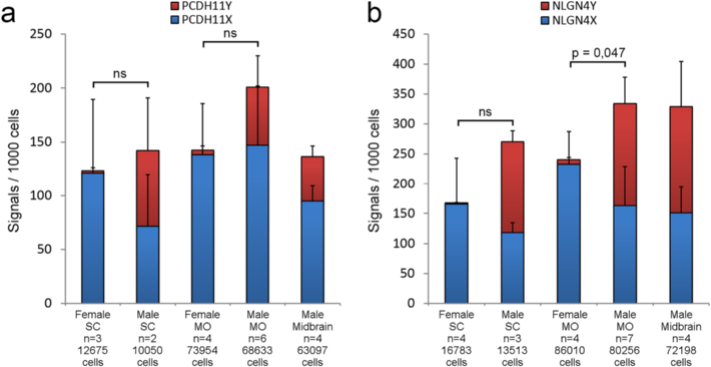
Quantification of padlock hybridization signals in SC and MO. The figure shows the average amount of signals per 1000 cells, identified by the image analysis software CellProfiler in the original microscopy images, for female and male SC, MO and midbrain. Standard errors are included for both females and males. The number of sections and the total number of cells analysed are indicated under each staple bar. **a**. PCDH11X/Y. **b** NLGN4X/Y. The *clamps on top* of the bars indicate the significance (or not) of the comparison between X gene expression between females and males

We used the padlock probes designed as described in the “Methods” section, as a cocktail for transcript detection by padlock probe ligation and RCA on sections prepared from male and female embryonic CNS tissues. Due to sample scarcity of female brain samples, we decided to focus on the MO and spinal cord (SC) for the *in situ* analysis. We observed that while the X-specific padlock probes detected transcripts in both female and male samples, the Y-specific padlock probes almost exclusively detected transcripts in male tissue sections, with very low background hybridization to females, confirming the sex specificity of the Y-specific padlock probes (Fig. 2). The low number of signals for the Y homologs, observed in the female tissues, were likely due to mis-ligation events of the Y-specific padlock probes but even more likely due to false-positive signals (autofluorescent rolling circle product like signals) identified by the image analysis software CellProfiler. In addition, we observed that both PCDH11X/Y and NLGN4X/Y mRNA expression are mainly confined to the grey matter, with very little expression in the white matter (“Wm” in Fig. 2g–j). As the samples are not comparable with respect to absolute signal counts, the counts for X and Y signals were normalized to counts per 1000 cells. When we quantified the amount of *PCDH11X* and *PCDH11Y* signals in 7 sections obtained from female SC tissues and 12 sections from male tissue, we observed that the amount of signals (per 1000 cells) for *PCDH11X* did not differ between male and female in both the SC and MO (Fig. 3), in accordance with the results from the RNA sequencing of the MO. On average, 50 % of the detected transcripts in the male SC corresponded to Y transcripts, and 50 % to X transcripts. In the male MO, the proportions were 27 % Y and 73 % X. When we quantified NLGN4X/Y signals in 8 sections obtained from female tissues and 14 sections from males (Table 2), we did not find differences in the number of observed signals for *NLGN4X* transcripts in females compared to males in both tissue types. On average, 56 % of the observed transcripts in male SC were Y transcripts, and 44 % were X transcripts. In the MO, the proportions were 51 and 49 %. These results correlated well with expression levels measured by RNA sequencing.

### Cell classification based on transcriptional profiles

**Fig. 4:**
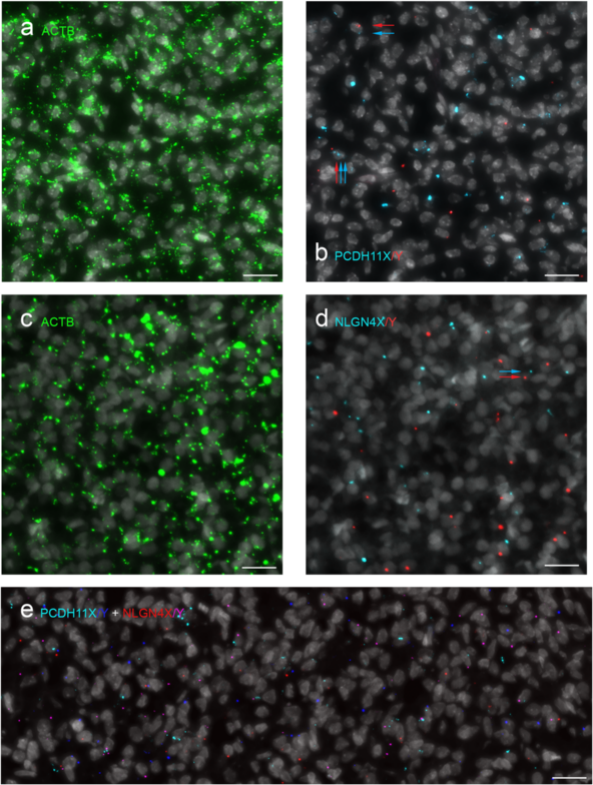
Padlock hybridization with *ACTB*, PCDH11X/Y and NLGN4X/Y. **a**, **b**. Male MO sections simultaneously hybridized with one padlock probe for *ACTB*, five probes for *PCDH11X* and five for *PCDH11Y*. **a**, **b** The same region of the section with staining for nuclei and ACTB (**a**) and *PCDH11X* (*cyan*) and *PCDH11Y* (*red*) (**b**). Most stained cells express either *PCDH11X* or *PCDH11Y*. Rarely, some cells express both X and Y transcripts (marked with *arrows*). **c**, **d** Male MO section simultaneously hybridized with one padlock probe for *ACTB*, four probes for *NLGN4X* and four for *NLGN4Y*. Nuclei and *ACTB* staining are shown in **c**, and *NLGN4X* (*cyan*) and NLGN4Y (*red*) staining in **d**. **e** Male SC section simultaneously hybridized with five probes for *PCDH11X* (*cyan*), five for *PCDH11Y* (*blue*), four probes for *NLGN4X* (*red*) and four for *NLGN4Y* (*purple*)

Figure 4 shows the distribution of PCDH11X/Y, NLGN4X/Y, and *ACTB* (housekeeping gene for tissue quality control) signals in male tissues. We observed that signal-positive cells for both PCDH11X/Y and NLGN4X/Y were in most cases positive for X or Y transcripts, but not for both. Examples of these cells are marked with arrows in Fig. 4b, d. To determine whether cells were expressing the X and the Y homologs in a mutual exclusive fashion, we classified cells with at least two assigned signals as X-specific, Y-specific or mixed. Based on this classification (Tables  1 and 2), we observed that for both gene homolog pairs, there were comparable numbers of cells in all three cell classes, indicating that cells can simultaneously express X and Y transcripts as well as exhibit a mutually exclusive fashion of expression.

**Table 1:** Distribution of cells with PCDH11X/Y transcripts: amount of cells with PCDH11X/Y signals

Sample	Tissue	Gene	Gender	Cells without signals	Cells with X = 1	Cells with Y = 1	Cells with X + Y > = 2	Cells with X > 1 Y = 0	Cells with Y > 1 X = 0	Total cell counts
E27_S44	SC	PCDH11	Female	3442	470	1	0	89	0	4002
E31_S91	SC	PCDH11	Female	3358	190	4	0	23	1	3576
E49_S82	SC	PCDH11	Female	4793	252	18	0	34	0	5097
E33_S50	MO	PCDH11	Female	13,517	1479	10	6	321	0	15,333
E34_S20	MO	PCDH11	Female	12,485	1069	0	0	192	0	13,746
E33_S63	MO	PCDH11	Female	16,189	1042	45	1	113	0	17,390
E34_S63	MO	PCDH11	Female	25,784	1452	85	13	151	0	27,485
E32_S53	SC	PCDH11	Male	4915	106	61	10	5	14	5111
E32_S84	SC	PCDH11	Male	4319	257	269	45	31	18	4939
E45_S75	MO	PCDH11	Male	14,214	895	427	108	87	28	15,759
E61_S49	MO	PCDH11	Male	5944	636	78	21	122	2	6803
E41_S58	MO	PCDH11	Male	6824	409	99	13	41	0	7386
E45_S50	MO	PCDH11	Male	16,881	1553	948	240	175	85	19,882
E58_S82	MO	PCDH11	Male	9437	1122	356	160	241	15	11,331
E61_S72	MO	PCDH11	Male	6848	373	194	17	34	6	7472
E47_S71	Midbrain	PCDH11	Male	9635	683	172	58	53	9	10,610
E45_S330	Midbrain	PCDH11	Male	15,907	595	369	43	62	17	16,993
E47_S72	Midbrain	PCDH11	Male	8758	468	135	18	47	7	9433
E45_S329	Midbrain	PCDH11	Male	24,012	1259	555	70	137	28	26,061

Summary of signal and cell counts from the padlock probing *in situ* experiments. The table shows the total number of signals for each gene, and the total number of cells identified by CellProfiler, for each sample hybridized simultaneously with padlock probes for two X and Y genes

**Table 2:** Distribution of cells with NLGN4X/Y transcripts: amount of cells with NLGN4X/Y signals

Sample	Tissue	Gene	Gender	Cells without signals	Cells with X = 1	Cells with Y = 1	Cells with X + Y > = 2	Cells with X > 1 Y = 0	Cells with Y > 1 X = 0	Total cell counts
E27_S48	SC	NLGN4	Female	3098	364	3	4	45	0	3514
E31_S56	SC	NLGN4	Female	2955	493	3	0	94	1	3546
E49_S81	SC	NLGN4	Female	4225	350	4	0	47	0	4626
E49_S82	SC	NLGN4	Female	4767	297	9	1	23	0	5097
E33_S62	MO	NLGN4	Female	12,552	1917	20	4	284	0	14,777
E34_S61	MO	NLGN4	Female	22,028	3586	40	25	674	5	26,358
E33_S63	MO	NLGN4	Female	15,497	1652	49	14	177	1	17,390
E34_S63	MO	NLGN4	Female	24,119	2871	76	33	381	5	27,485
E26_S65	SC	NLGN4	Male	3165	200	303	36	26	25	3755
E32_S29	SC	NLGN4	Male	3917	319	447	75	25	36	4819
E32_S84	SC	NLGN4	Male	4120	310	381	69	28	31	4939
E61_S39	MO	NLGN4	Male	5283	379	414	58	19	47	6200
E61_S89	MO	NLGN4	Male	6123	633	710	117	56	75	7714
E45_S48	MO	NLGN4	Male	16,555	1564	1580	295	133	144	20,271
E41_S58	MO	NLGN4	Male	5719	563	584	421	50	49	7386
E45_S50	MO	NLGN4	Male	16,356	1644	1261	391	155	75	19,882
E58_S82	MO	NLGN4	Male	8219	1205	1102	491	200	114	11,331
E61_S72	MO	NLGN4	Male	6405	273	645	68	19	62	7472
E45_S329	Midbrain	NLGN4	Male	24,917	1623	2226	292	107	153	29,318
E47_S77	Midbrain	NLGN4	Male	5774	558	772	165	50	67	7386
E45_S329	Midbrain	NLGN4	Male	21,915	1678	1771	389	174	134	26,061
E47_S72	Midbrain	NLGN4	Male	8124	596	559	64	55	35	9433

Summary of signal and cell counts from the padlock probing *in situ* experiments. The table shows the total number of signals for each gene, and the total number of cells identified by CellProfiler, for each sample hybridized simultaneously with padlock probes for two X and Y genes

To evaluate how PCDH11X/Y and NLGN4X/Y were expressed in relation to each other, we hybridized sections simultaneously with the four sets of padlock probes using a four colour detection system as described in the “Methods” section (Fig. 4e). The analysis of single-cell expression profiles shows all possible combinations of the four transcripts (Table 3).

**Table 3:** Distribution of cells with simultaneous detection of PCDH11X/Y and NLGN4X/Y transcripts

Sample ID	Tissue	Gene	Gender	Total cell counts	Cells with PX > = 1	Cells with PY > = 1	Cells with NX > = 1	Cells with NY > = 1	Cells with PX + Y > = 2	Cells with NX + Y > = 2	Cells with PX + NX > = 2	Cells with PY + NY > = 2
E49_S82	SC	Dual	Female	5097	247	16	282	6	0	1	38	2
E33_S63	MO	Dual	Female	17,390	993	36	1671	39	1	12	153	4
E34_S63	MO	Dual	Female	27,485	1292	71	2940	68	5	25	302	2
E32_S84	SC	Dual	Male	4939	208	241	278	353	32	49	31	16
E41_S58	MO	Dual	Male	7386	318	75	566	573	8	367	39	15
E58_S82	MO	Dual	Male	11,331	887	241	1108	977	100	361	207	50
E61_S72	MO	Dual	Male	7472	322	155	245	628	13	60	31	27
E45_S329	Midbrain	Dual	Male	26,061	1084	447	1625	1714	46	335	142	42
E47_S72	Midbrain	Dual	Male	9433	421	107	591	530	15	56	42	16
Sample ID	Tissue	Gene	Gender		Cells with PX + NY > = 2	Cells with PY + NX > = 2	Cells with PX + NX + NY > = 3	Cells with PY + NX + NY > = 3	Cells with PX + PY + NX > = 3	Cells with PX + PY + NY > = 3	Cells with PXandY + NXandY > = 4	Cells with no signals
E49_S82	SC	Dual	Female		1	0	0	0	0	0	0	4504
E33_S63	MO	Dual	Female		7	5	2	0	2	0	0	14,467
E34_S63	MO	Dual	Female		5	9	4	3	4	3	1	22,751
E32_S84	SC	Dual	Male		38	23	11	7	11	7	2	3639
E41_S58	MO	Dual	Male		43	5	50	4	50	4	0	5318
E58_S82	MO	Dual	Male		169	58	100	22	100	22	8	6991
E61_S72	MO	Dual	Male		50	15	4	3	4	3	1	5915
E45_S329	Midbrain	Dual	Male		141	74	29	20	29	20	5	20,338
E47_S72	Midbrain	Dual	Male		46	17	6	2	6	2	0	7581

The table shows the total number of signals for each gene, and the total number of cells identified by CellProfiler, for each sample hybridized simultaneously with padlock probes for the four X or Y genes: *PCDH11X* (PX), *PCDH11Y* (PY), *NLGN4X* (NX) and *NLGNY* (NY)

### Differential spatial distribution of X and Y expressing cells in the CNS

**Fig. 5:**
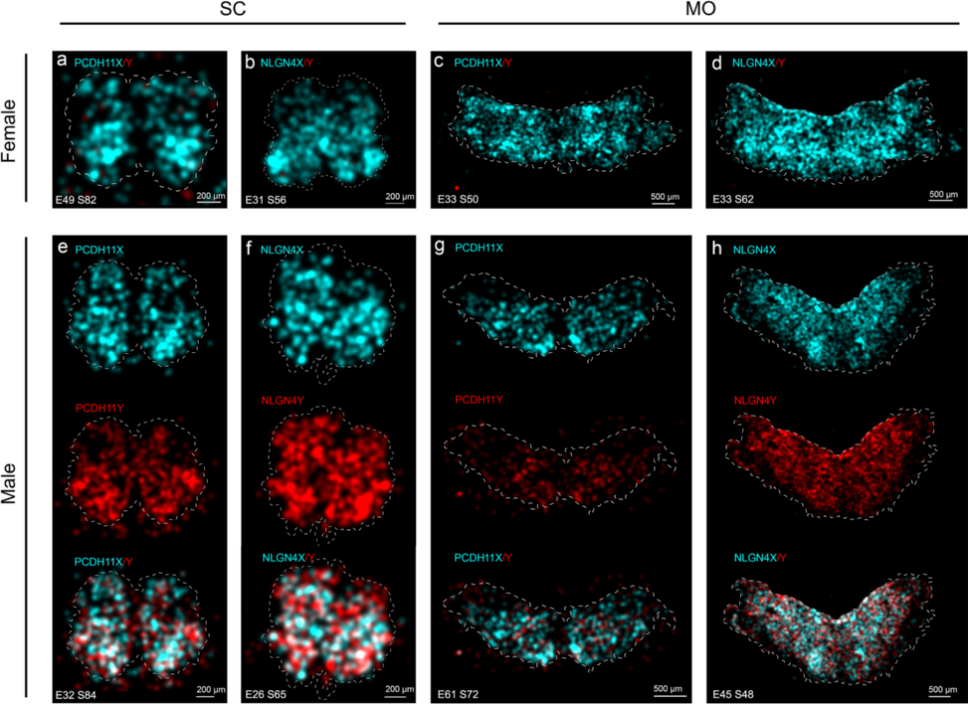
Signal density maps in the spinal cord and medulla oblongata sections from female and male embryos hybridized with PCDH11X/Y and NLGNX/Y. The distributions of Y and X signals in different tissue sections are displayed as kernel density estimation plots where Y signal density is displayed in *red* and X signal density in *cyan*. The intensity of the colour reflects the density of the signal. Overlap in signal densities results in a mixed colour, whereas equal proportion of Y and X results in white colour. The outline of the tissue is indicated by a *dashed white line*. Orientation of the SC tissues is ventral down and dorsal up. The signal density in the female spinal cord is displayed in **a** for *PCDH11X* and in **b** for *NLGN4X*. **c**, **d** Signal density in the female medulla oblongata for *PCDH11X* and *NLGN4X*, respectively. **e** Signal density of PCDH11X/Y in the male spinal cord is shown individually for the two homologs as well as a plot of the two transcript densities combined. NLGN4X/Y is displayed in the same way on **f**. Signal densities in the male medulla oblongata are shown in **g** and **h** for PCDH11X/Y and NLGN4X/Y

To conduct an analysis of the spatial distribution patterns and objectively investigate whether transcripts for *PCDH11X*, *PCDH11Y*, *NLGN4X* and *NLGN4Y* are differentially distributed in the CNS as well as to visualize the extent of overlap of areas with X and Y signals, we compared our image data with a randomized image data set. As a first step, with these three goals in mind, we generated kernel density estimation plots as described in the “Methods” section and plotted the X and Y signal densities onto images of the SC and MO tissue sections (Fig. 5). In the SC, the signal density of both *NLGN4X* and *PCDH11X* varied across each tissue section, with clearly higher signal density in the ventral horns than in the dorsal horns in both male and female tissues (Fig. 5a, b, e–f). In contrast to the X homologs, *NLGN4Y* presented an even distribution over the whole SC, suggesting that there are cells which are more prone to express the Y homolog than the X homolog in some parts of the sections, namely in the dorsal horns (Fig. 5f). *PCDH11Y* did not show any consistent signal distribution differences between the ventral and dorsal regions (Fig. 5e). Different expression patterns were also observed when we analysed sections from the MO (Fig. 5c, d, g–h). Indeed, density plots showed how *PCDH11X* appeared to be expressed patch-wise in both male and female MO tissues while *PCDH11Y* showed a more evenly distributed expression pattern, to a larger extent than for *PCDH11X* confined to the periphery of the tissue. Similarly, the *NLGN4Y* transcripts appeared to be homogeneously distributed over the tissue sections, while the density of *NLGN4X* transcripts varied across the tissues.

**Fig. 6:**
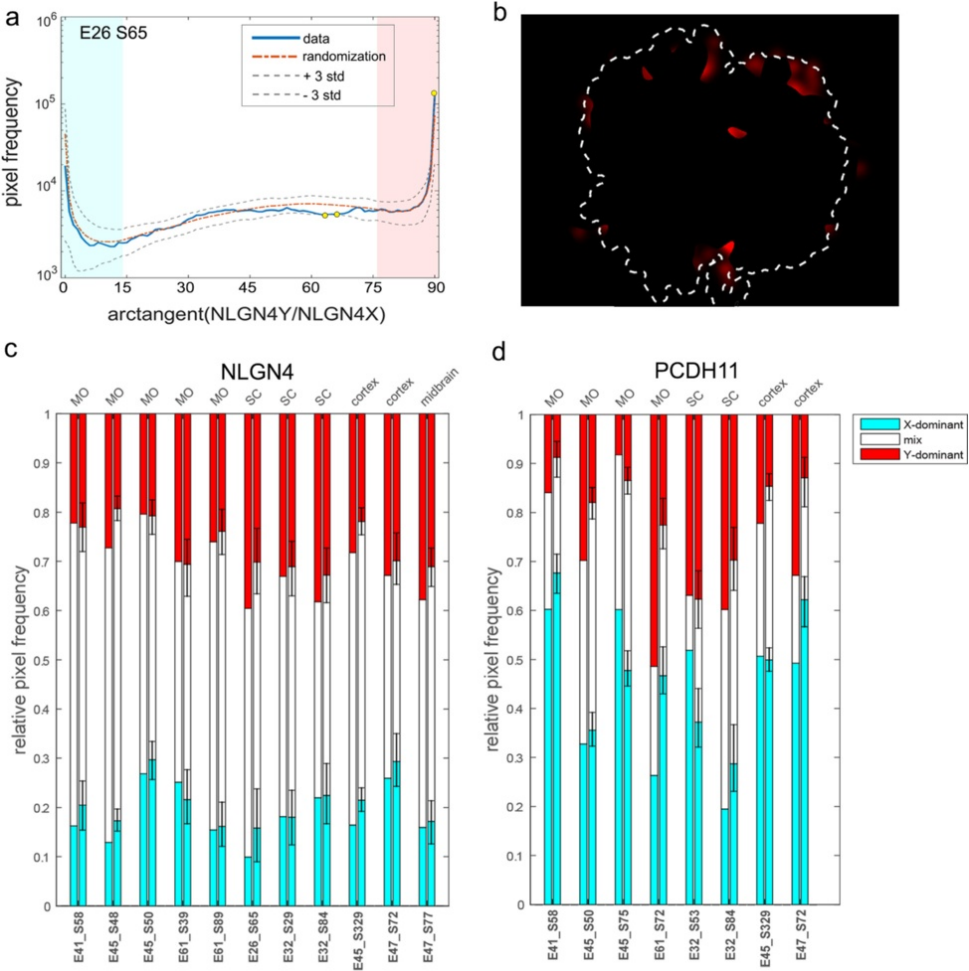
Comparison of the observed spatial signal distribution to randomized signal distributions in male MO for NLGN4X/Y. The frequency of pixels of varying intensity ratios of X and Y for NLGN4X/Y in male SC (E32_S84) are displayed in the *histogram* in **a**. A cutoff of 80 % colour purity was set to classify pixels as X dominant and Y dominant, displayed in the histogram as a *blue field* and a *pink field*, respectively. Pixels deviating more than 3 SD are shown as *yellow dots* on the *blue line* and then plotted in **b** visualize the location of the pixels over-represented in the sample tissue compared to the randomized data set. The proportions of X-dominant, Y-dominant and mixed pixels in the analysed samples are shown for all samples next to their respective randomized data set in **c** for NLGN4X/Y and in **d** for PCDH11X/Y. Relative pixel frequency from the random distributions for all samples is shown in **c** and **d**.

We then compared the observed data for male samples with a randomized data set and plotted the pixels whose colour is over-represented by more than 3 standard deviations compared to the mean value from 100 randomizations as described in the “Methods” section. For *NLGN4X* and *Y*, we see a deviation in the representation of Y-dominant pixels from the random data. X-dominant pixels are generally under-represented while Y-dominant pixels are over-represented in our data (Fig. 6a) in comparison to the randomized data set. This is consistent with the observation that the expression of *NLGN4X* is confined to some regions while *NLGN4Y* is more universally expressed. We also see tendencies to an under-representation of mixed pixels. When plotting the pixels deviating more than +3 SD, we observed a higher prevalence of *NLGN4Y* transcripts in the peripheral parts of the SC (Fig. 6b). An additional example is given in Additional file 2: Figure S1. For *PCDH11* in the MO, we observe high signal density for both *PCDH11X* and Y in the olivary nucleus (OL), but we also see an under-representation of pixels of mixed intensities, pointing towards a compartmentalized expression of *PCDH11X* and *PCDH11Y*. When comparing the analysed samples to their respective randomized data with 95 % confidence intervals (corresponding to a significance level of 0.05), we clearly see an over-representation of *NLGN4Y-*dominant pixels in the SC and brain samples, while in the MO, the trend is less clear and the difference between the data and randomized simulations is not statistically significant (Fig. 6c). We do not observe any consistent pattern for *NGLN4X* across all samples. For *PCDH11X/Y*, a majority of the samples show a strong deviation from randomness, and again, Y-dominant pixels are over-represented and no stable pattern is found for X-dominant pixels (Fig. 6d).

When comparing the ventral and dorsal counts for the homolog transcripts in spinal cord as the ratio between X and Y transcripts (Additional file 3: Table S2), we found that *NLGN4X* in females showed higher expression in the ventral SC (*n* = 5, *t* test, one tail, *p* = 0.045) compared to the dorsal. *PCDH11X* did not exhibit any significant differences in regional expression in females (*n* = 2, *t* test, one tail, *p* = 0.08). Neither *NLGN4Y* nor *PCDH11Y* presented significant differences in the dorsal to ventral expression patterns in males. This confirms the results from the analysis of the Kernel density estimation (KDE) plots, as the over-representation of Y-dominant pixels (located in the dorsal region) is expected when the Y homolog transcripts are more homogeneously distributed and the X homolog transcripts largely confined to the ventral region. Also, under-representation of X-dominant pixels is expected for the clustered expression pattern of the X homolog transcripts as these to a large extent overlap with the Y transcript signals resulting in mixed pixels in the ventral region. These observations suggested to us that there exist different populations of cells in the CNS with respect to the expression of X or Y homologs. In other words, our results indicate that spatial sexual dimorphism in the expression of these two genes exists.

### Determination of the cellular identity of Y expressing cells using a combination of immunohistochemistry with padlock probing

**Fig. 7:**
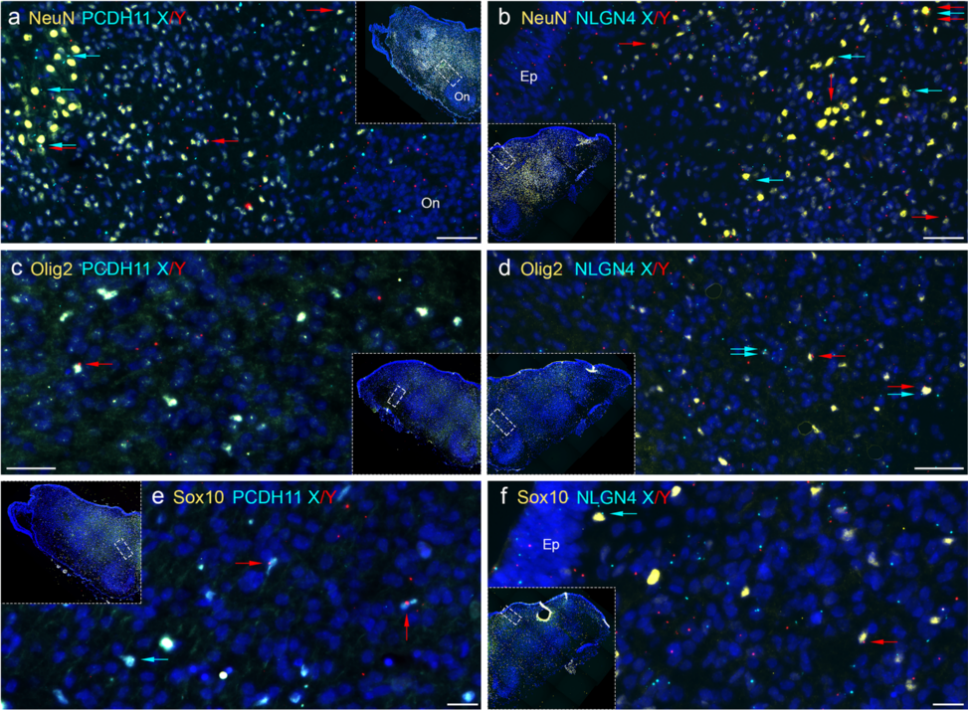
Combined padlock hybridization and immunohistochemistry for PCDH11X/Y and NLGNX/Y in MO samples from human male embryos. **a**, **b** Padlock probe hybridizations with probes for *PCDH11X* and *PCDH11Y* (**a**) or *NLGN4X* and *NLGN4Y* (**b**) were combined with immunohistochemistry using NeuN antibodies. NeuN-positive cells are stained in *yellow*, *PCDH11X* signals in *sky blue* and *PCDH11Y* in *red*. DAPI staining in dark blue allows visualization of nuclei. Each subfigure is an enlargement of the MO tissue showed in each *inset picture*. Some *NeuN*+ cells contain signals for *PCDH11X* (marked with *blue arrows*), *PCDH11Y* (*red arrows*) or both X and Y transcripts (*blue and red arrows*). **c**, **d** Padlock probe hybridization with probes for PCDH11X/Y (**a**) or NLGN4X/Y (**b**) was combined with immunohistochemistry using Olig2 antibodies. **d**, **f** Padlock probe hybridization with probes for PCDH11X/Y (**a**) or NLGN4X/Y (**b**) was combined with immunohistochemistry using Sox10 antibodies. *Ep* ependymal layer

To determine the precursor, glial or neuronal identity of positive cells for PCDH11X/Y and NLGN4X/Y in the MO, we combined padlock probe hybridizations with immuno-histochemical labelling as described in the “Methods” section. We used NeuN antibodies, known to label neurons [28], Olig2 antibodies, which stain precursor cells for oligodendrocytes [29], and Sox10 antibodies, which stain neural progenitor cells which will give rise to both neural and glial cells in the CNS [30]. Figure 7a shows that both NeuN+ and NeuN− cells express *PCDH11X* and *PCDH11Y* transcripts. For example, the olivary nucleus (ON) region contains *PCDH11X* and Y signals but no NeuN staining. Similarly, Fig. 7b shows also a partial overlap between *NLGNX* and Y signals and NeuN staining. Interestingly, *NLGN4X* and Y signals were observed in the ependymal layer (“Ep” in Fig. 7b) while signals for *PCDH11X* and Y were sparse in this region (not shown). Ep is the layer from which glial cells originate and migrate [31]. Olig2 staining is shown in Fig. 7c, d. Only a small subset of Olig2+ cells and Sox10+ cells contained signals for *PCDH11X*, *PCDH11Y*, *NLGN4X* or *NLGN4Y*, suggesting partial expression of X and Y gametolog genes in neuronal cell precursors (Fig. 7e, f). In addition to labelling precursors for oligodendrocytes, Olig2 is known to be expressed in motor neurons [29]. Furthermore, as described in the section related to density map analysis above, we observed increased expression of *PCDH11X* and *NLGN4X* in the ventral horn of the SC, a region known to contain several nuclei for motor neurons. Therefore, we also performed combined padlock probing with immunostaining for *Islet-1* antibodies, known to label motor neurons, in the middle and ventral horn of the SC [32]. Additional file 4: Figure S2 shows that we found co-localization of *PCDH11X* and Y signals and Islet-1 in some positive cells. This suggests that *PCDH11X* and Y are expressed more in motor neuron differentiating cells during early stages of development than in sensory neurons, known to occupy the dorsal horn of the SC [33]. In conclusion, both *PCDH11X* and Y and *NLGNX* and Y are expressed in heterogeneous sub-populations of cells at different developmental states, including developing neurons and glial precursors.

## Discussion

This study provides the first evidence of sexually dimorphic spatial expression patterns of Y chromosome genes and their X-encoded homologs in the CNS tissues of human embryos during early development. We also show that X and Y transcripts can be simultaneously expressed in single cells and that a fraction of cells express the X or the Y homolog alone. Furthermore, the combined results from padlock probe hybridizations and RNA sequencing showed that the sum of gametolog gene expression in males is similar to the expression of the X homolog gene in females. Taking both main observations together, we can conclude that while the total expression of the gametolog genes is similar among the sexes, sex specificity in the developing male SC and MO may be due to the fact that Y transcripts are expressed in different sub-populations of cells (possibly cellular networks) than those expressing X transcripts in females. However, our results suggest but do not prove that X and Y transcripts can be expressed in different cells or that there exist different cell populations expressing the X and the Y homologs in different proportions.

The *PCDH11X* and Y genes are not the only members of the protocadherin family that showed combinatorial expression in different cells. In fact, eight protocadherins and five classical cadherins were expressed in a combinatorial way in cells of the primary somatosensory cortex of the adult mouse [34]. The authors showed that most single neurons express more than one cadherin in different combinations in all layers of cerebral cortex. Also, as in our study, some of the cadherin-positive cells were NeuN positive while others were not, indicating both neuronal and possibly glial expression of these family members. They proposed that the combinatorial expression of multiple cadherin genes provides a molecular code that contributes to the molecular specification of the vast complexity of cellular networks and connectivity in the brain. Our results suggest that an additional male-specific code may act in combination with the above, resulting in a subset of male-specific cellular networks or connectivity.

The specific functions of *PCDH11Y* and *NLGN4Y* cannot be studied by knockout experiments in mice, since rodents do not have these genes encoded in their genomes. On the other hand, the homolog X genes are present on rodents. Knockout mice for *Pcdh11x* have not yet been produced. However, in utero electroporation of embryos with short hairpin RNA (shRNA) or overexpression vectors against *Pcdh11x*, as well as transient transfection of neural stem cells collected from E13.5 mice embryos has been done recently [35]. These experiments showed that overexpression of Pcdh11x in embryonic neural stem cells leads to reduced neural stem cell differentiation, as well as enhanced neural proliferation, both in vivo and in vitro, while reduction of *Pcdh11x* resulted in increased cell migration and premature neuronal differentiation in mice. Very recently, it was shown that overexpression of *Pcdh11x* in primary cultures of cortical neurons derived from embryonic day 16 mice reduced dendritic complexity, whereas knockdown increased dendritic branching [36]. Assuming that the human *PCDH11Y* has similar functions as the X gene has in mice, then the expression of this gene only in human males could result in enhanced neural cell proliferation only in males and only in a subset of cell populations. A fundamental question for the future is whether the newly discovered subset of human male cells that express *PCDH11Y* (and not *PCDH11X*) are also present in females or whether they form a male-specific cellular network produced only in males during CNS development.

The effect of the removal of the homolog X-encoded gene has also been studied for *Nlgn4x*. Mice with a loss-of-function mutation in the murine ortholog *Nlgn4x*, which encodes the synaptic cell adhesion protein neuroligin-4, exhibited highly selective deficits in reciprocal social interactions and communication [37], although later generations of the same line of *Nlgn4* mutant mice did not show these behaviours [38]. Also, absence of *Nlgn4x* caused a decreased network response to stimulation in both excitatory and inhibitory circuits in somatosensory cortical slices of juvenile mice [39].

In humans, multiple genetic studies have implicated a significant role for *NLGN4X* in the susceptibility to several neurodevelopmental disorders. For example, Xp22.3 deletions including *NLGN4X* have long been associated with autism [40]. Also, a frameshift mutation in *NLGN4X* causing a premature stop codon was observed in all affected individuals in a large family, in which 10 males had X-linked mental retardation, 2 had autism, and 1 had pervasive developmental disorder [41]. *PCDH11X* is also proposed to affect neurodevelopment in humans. For example, deletion of this gene was found in a patient with developmental dyslexia [42], and deletion of both *PCDH11X* and *PCDH11Y* was discovered in a child with non-syndromic language delay [6]. Despite these prior human genetic studies, it is still not known what biological processes and cellular mechanisms are compromised when mutations in *PCDH11X* or *NLGN4X* occur. As mentioned before, functional studies of these genes can only partially be addressed in animal models. Therefore, it seems that functional insight in humans, particularly during neurodevelopment, will be best addressed by the use of human pluripotent stem cell (hiPSC) approaches [43]. Interestingly, neural stem cell (NSC) models derived from both hiPSCs and human embryonic stem cells have recently been used to study the function of *NLGN4X* [44]. The authors used shRNAmir to repress NLGNX expression. They observed that decreased *NLGN4X* levels during differentiation of NSCs into neurons caused delayed neuronal development, decreased neurite formation and less cell–cell connections.

## Conclusions

The main findings of our investigations were that the X and Y homolog genes for PCDH11 and NLGN4 were mostly expressed in different glial and neuronal cell populations in the CNS during human male development. Also, we found differential distribution patterns of X and Y homologs in the male developing central nervous system. We propose that future experiments on NSC models derived both from females and males in which X and Y homolog genes are differentially modulated will shed light on the possible involvement of these genes in the development, maturation or connectivity of sex-specific cellular networks in the human brain.

## Methods

### Human embryonic CNS samples and sample preparation

Samples were obtained from human embryos between the 7 and 11 gestational weeks. The gestational age was estimated from the last menstrual period and from an ultrasound scan in which crown-rump length of the foetus was measured. Samples were dissected after surgical terminations of pregnancies performed at the University Hospital in Uppsala and after maternal written consent and approval from the regional Ethics Committee in Uppsala (number 2011/329). All procedures were made in general anaesthesia by intravenous administration of 1 ml alfentanil 0.5 mg/ml and 15–20 ml propofol 10 mg/ml. No gases for inhalation were used during the process, except for a mix of air and oxygen. The short procedure for evacuating the uterine cavity was performed by a gynaecologist with vacuum aspiration. The tissue specimens were disrupted by the surgical procedure, and the tissue types most frequently intact were, in declining order, the MO, SC and midbrain. Prior to sectioning, tissue samples were fixed for at least 16 h at 4 °C in 0.1 M phosphate-buffered saline (PBS) containing 4 % paraformaldehyde (PFA; Sigma-Aldrich). The samples were then cryoprotected in 30 % sucrose/PBS. Twelve-micrometre-thick sections were cut on a freezing microtome after mounting in TissueTec® (check name), collected on SuperFrost® Plus microscope slides (VWR International), and stored at −80 °C prior to hybridizations. The samples for RNA sequencing were snap frozen on dry ice - chilled Eppendorf tubes and stored at −80 °C prior to RNA extractions. To determine the sex of each embryo, DNA was extracted from arm tissue using DNasy® Blood & Tissue Kit (Qiagen). PCR on DNA samples was done using male-specific primers for STS sY14 (SRY), (5′-GAATATTCCCGCTCTCCGGA-3′, 5′-GCTGGTGCTCCATTCTTGAG-3). Complete information about the samples used for each experiment can be found in Additional file 5: Table S3.

### Preparation of RNA-seq libraries from total and polyA+ RNA

Frozen tissues ranging from 70–190 mg were homogenized in 2 ml TRIzol® reagent (Ambion) using an Ultratorrax T25 homogenizator (Labortechnik). Total RNA was extracted using RiboPure kit (Ambion) according to the manufacturers’ instructions. PolyA RNA was enriched from 1 μg total RNA using MicroPoly (A) Purist kit (Ambion) according to the manufacturer’s instructions. The quantity and quality of the input RNA was controlled using a RNA 6000 Pico chip on a Bioanalyzer (Agilent Technologies) and only RIN values above 7 were used in the analysis.

Complementary DNA (cDNA) library preparation was conducted at the Uppsala Genome Centre (SciLifeLab). Briefly, a ribosomal RNA (rRNA) depletion step was performed with 56 mg as input amount for all samples, using the RiboMinus Eukaryote kit (Life Technologies). Whole transcriptome libraries were then constructed using the SOLiD total RNA-Seq kit (rev B, July 2011, Life Technologies). Emulsion PCR was performed using the SOliD EZ Bead System (Life Technologies) and the libraries were then sequenced on three lanes with the SOLiD 5500xl System (Life Technologies).

### Sequence alignment and data analysis

All sequencing reads were aligned to the hg19 build of the human genome using the LifeScope software (version 2.5, Life Technologies). Reads mapping to exonic regions were counted using the HTSeq software (http://www-huber.embl.de/users/anders/HTSeq/doc/overview.html) only counting reads having the quality of 30 or higher (Additional file 6: Figure S3) using the hg19 gene list (gff file) provided from the iGenomes project (Illumina) [45]. Differential gene expression was calculated using the DESeq2 software (version 1.6.0) [46]. DESeq2 approximates the null distribution using a negative binomial model under the assumption that the two conditions have the same read abundance, in order to calculate the probability of genes having a differential expression between two samples. Correction for multiple testing was done by DESeq2 using the procedure of Benjamini and Hochberg [45]. When counting reads aligned to specific exons used for design of padlock probes, the HTseq software was used as previously described, using the specific exons as input. RPKM calculations were performed using the R RPKM library. Reads overlapping each padlock probe were counted using SAM tools together with in-house perl scripts.

### Sample pre-treatment for *in situ* experiments

Tissue samples were dried at room temperature for 1 h prior to two washes in 0.1 % diethylpyrocarbonate (DEPC)-treated PBS. The tissue was then permeabilized in 0.2 mg/ml pepsin (Sigma) in 0.1 M HCl at 37 °C for 2 min. After permeabilization, the slides were washed twice in DEPC-treated PBS. All of the following reactions were performed in Secure Seal hybridization chambers (Invitrogen).

### Padlock probe design strategy

Alignments of transcripts for *PCDH11X* and *PCDH11Y* showed very high levels of sequence identity between these genes (Additional file 7: Figure S4a), with 97 to 99 % sequence identity for each exon (Additional file 8: Table S4a). Similarly, alignments for *NLGN4X* and *NLGN4Y* presented 89 to 98 % identity at the exon level (Additional file 7: Figure S4b and Additional file 8: Table S4b). We selected two positions in exon 5 of *PCDH11X/Y* to design padlock probes P1 and P2 (Additional file 7: Figure S4a). These positions differ by one nucleotide in all X transcripts compared to all Y transcripts. In addition, we selected three positions in exon 9 which differ between all *PCDH11X* long transcripts (NM_032968, NM_032969, NM_001168360, NM_001138361, NM_001138362, NM_001138363), compared to the only known *PCDH11Y* long transcript (NM_032973), and we designed probes P3, P4 and P5. Similarly, for *NLGN4X/Y*, we selected one position in exon 3, one in exon 5 and 2 positions in exon 6, all of them present in all X and Y transcripts, to design padlock probes N1 to N4 (Additional file 7: Figure S4b). The complete sequences of all padlock probes including cDNA primers and detection sequences are listed in Additional file 9: Table S5. All padlock probes were 5′-phosphorylated at a concentration of 2 μM with 0.2 U μl^−1^ T4 polynucleotide kinase (Fermentas) in the manufacturer’s buffer A plus 1 mM ATP for 30 min at 37 °C, followed by enzyme inactivation for 10 min at 65 °C.

### *In situ* cDNA detection using padlock probes

To start the reverse transcription step, 1 μM of each cDNA primer was added to the slides with 20 U μl^−1^ of RevertAid H minus M-MuLV reverse transcriptase (Fermentas), 500 nM dNTPs (Fermentas), 0.2 μg μl^−1^ BSA (NEB) and 1 U μl^−1^ RiboLock RNase Inhibitor (Fermentas) in the M-MuLV reaction buffer. The slides were incubated for 3 h at 37 °C. After incubation, the slides were washed briefly in PBS-T (DEPC-PBS with 0.05 % Tween-20 (Sigma)), followed by a post-fixation step in 3.7 % (*w*/*v*) paraformaldehyde in DEPC–PBS for 45 min at room temperature. Subsequently, the samples were washed twice in PBS-T.

To make the target cDNA strands available for padlock probe hybridization, the RNA portion of the created RNA–DNA hybrids was degraded with ribonuclease H. This was performed in the same step as the padlock probe hybridization and ligation. Ligation was carried out with 100 nM of each padlock probe in a mixture of 0.5 U μl^*−*1^ Tth DNA Ligase (Gene Craft Germany), 0.4 U μl^−1^ RNase H (Fermentas), 1 U μl^−1^ RiboLock RNase Inhibitor, Tth ligase buffer, 50 mM KCl and 20 % formamide. Incubation was performed first at 37 °C for 30 min, followed by 45 min at 45 °C. RCA was performed with 1 U μl^−1^ Φ29 DNA polymerase (Fermentas) in the supplied reaction buffer, 1 U μl^−1^ RiboLock RNase Inhibitor, 250 μM dNTPs, 0.2 μg μl^−1^ BSA and 5 % glycerol. Incubation was carried out for 16 h at 37 °C. The incubation was followed by a wash in PBS-T.

RCA products were visualized using 100 nM of each corresponding detection probe (Additional file 9: Table S5) in 2× SSC and 20 % formamide at 37 °C for 30 min. Each detection oligonucleotide is available labelled with FITC, Texas Red (TR), Cy3, Cy5 or AF750. For two colour detection of X and Y transcripts, we used Cy3 and TR, respectively. ACTB transcripts were detected using Cy5-labelled detection oligonucleotide. For simultaneous four colour detection of transcripts from four genes, we used Cy3-labelled detection oligonucleotides for PCDH11X, TR for PCDH11Y, AF750 for NLGN4X and Cy5 for NLGN4Y. For simultaneous four colour detection of splice variants, we used Cy3 for PCDH11X-PAN, Cy5 for PCDH11X-Long, FITC for PCDH11Y-PAN and TR for PCDH11Y-Long. The slides were then washed in PBS-T, and nuclei staining was done using Hoechst 33343 (Life Technologies) 50 μg μl^−1^ for 12 min at room temperature. Finally, the slides were washed with PBS-T followed by removal of the secure-seals, and the slides were dehydrated using a series of 70, 85 and 99.5 % ethanol for 2 min in each. The dry slides were mounted with SlowFade® Gold antifade reagent (Life Technologies).

### Image acquisition and processing

The tissue slides were scanned using a Zeiss Axio Imager.Z2 epi-fluorescence microscope (filter cubes in Additional file 10: Table S6) and the original tiles (each one field of view of the camera) exported as grayscale images for image processing using the image analysis tool CellProfiler (http://www.cellprofiler.org/). A customized pipeline was set up for each of the samples for identification of transcript signals and nuclei based on the fluorescent signals. The nuclei were segmented based on global Otsu thresholding and the cell borders defined by a fixed distance of 20 pixels from the nuclei border (Additional file 11: Figure S5). Furthermore, the transcript signals were identified by manual thresholding subsequent to top-hat filtering of the images and then assigned to a parent cell based on in which identified cell they were detected. The transcript signals were counted in the whole tissue to obtain total counts for comparison to the RNA sequencing data. The numbers of detected transcript signals were summarized as well as the data for each of the segmented cells and their assigned children signals to classify cells based on expression of X, Y or both X and Y. The coordinates of the transcript signals were used to construct density plots of the tissues for investigation of the regional distribution of X and Y transcripts of NLGN4 and PCDH11.

### Image analysis

To statistically analyse whether differences in signal density distributions of X and Y transcripts were of biological relevance, we compared the observed data for male samples with a randomized data set. An image of a randomized distribution of signals was generated for each sample using the same positions of the signal and the same number of X and Y transcript counts as in the observed data. KDE plots were generated for each sample and randomized image data. The KDE plots were generated from binary images where each signal is represented by 1 pixel. The pixels are convoluted with a rotational symmetric 2D Gaussian kernel of 150 pixels with a standard deviation of 25 pixels. The Y and X pixel intensity ratio was calculated for the density plots, and the frequency of these pixel ratios for each sample data was plotted in a histogram together with the Y and X pixel intensity ratio frequency from the corresponding randomized data set (±3 SD; 100 randomizations). Since we were particularly interested to see whether we could find indications of cell populations having preferential expression of either X or Y transcripts, we decided to divide our data into three ratio bins. A dominant X or Y pixel was defined as a pixel with 80 % colour purity for either the X or Y homolog, leaving the rest of the pixels in a mixed bin.

### Analysis of dorsal vs. ventral SC signal distribution

The definition of dorsal and ventral border in SC tissue was done by dividing the total distance from dorsal to ventral horn at one position on each side of the SC. These two points acted as basis for a horizontal border separating the dorsal from the ventral region. Signals in each part of the tissue were counted using ImageJ Cell Counter plugin. Statistical evaluation was done using two-sample *t* test and one tail and assuming unequal variances.

### Combined padlock hybridization and immunohistochemistry

To combine the padlock detection and immunohistochemistry, we first hybridized slides with padlock probes as described above. After that, the slides were washed three times for 10 min in PBS and blocked for 1 h in 0.25 % bovine serum albumin in PBS with 0.25 % Triton X-100. Each of the following primary antibodies: anti-NeuN (mouse, 1:1000; Abcam), anti-Olig2 (goat, 1:20; R&D Systems), anti-Sox10 (goat, 1:20; R&D Systems) and anti-Islet 1 (goat, 1:20; R&D Systems) was incubated overnight at 4 °C. After washing with PBS, the corresponding secondary antibodies were applied for 4 h at room temperature: Alexa 594 goat anti-rabbit, FITC goat anti-mouse, Alexa 488 donkey anti-goat and Alexa 597 goat anti-chicken (1:400; Life Technologies). Immunolabelled cells were mounted with Dako ultramount aqueous permanent mounting medium (Dako). Cell imaging was performed using epi-fluorescence microscope Ziess (Zeiss Axio Imager.Z2 epi-fluorescence microscope, filter cubes in Additional file 10: Table S6). The images were assembled in Photoshop CS6 (Adobe).

## Additional file

Additional file 1: Table S1. RNA sequencing results for all genes belonging to protocadherin and neuroligin families. The table shows expression values as base mean values for females and males, the position of each gene in the genome, fold change in positive values for male-biased genes and negative values for female-biased genes and *p* values adjusted for multiple testing as described in the “Methods” section. (DOCX 27 kb) Additional file 2: Figure S1. Example of the objective analysis of spatial expression image data. Kernel density estimation (KDE) plots of PCDH11X/Y transcript signals detected in embryonic brain tissue are shown in a. At the top, the KDE plot of *PCDH11X* (cyan) has been merged with the one of *PCDH11Y* (red). From the separate KDE plots, two clear features can be distinguished; the Y homolog is more evenly expressed over the whole tissue while the X homolog shows higher expression in confined regions, namely along the lower edge and at the tip to the right. The observed spatial distribution of X and Y signals is compared to a random distribution in the histogram b, based on the relative contribution of X and Y signal intensity in each pixel in the KDE plot. The distribution of pixels in the KDE plot is shown as a blue line together with the average of 100 randomized data sets as a red line. Pixels deviating in number from what would be expected by chance (±3 SD from averaged random) are shown as a yellow dots on the blue line. The bar chart in b shows that we have a higher number of pixels classified as Y-dominant than we have X-dominant pixels, confirming our observations in the KDE plots in a that some regions express the Y homolog to a higher extent than the X homolog. Pixels in the observed signal distribution deviating more than 3 standard deviations from the random signal distribution, are plotted back onto the tissue in c and further confirms the patterns of X and Y homolog expression observed in a. The Y homolog shows dominant expression along the upper edge of the tissue section while the lower edge of the tissue section is dominated by mixed pixels (signal contribution from both the homologs). (TIF 623 kb) Additional file 3: Table S2. Signal counts and statistical analysis for the ventral and dorsal part of the spinal cord. The table shows signal counts in dorsal vs. ventral SC sections acquired by using ImageJ software (Cell Counter plugin). Distributions for *PCDH11X*, *PCDH11Y*, *NLGN4X* and *NLGN4Y* are shown for slides obtained from seven female and four male embryos. Percentage distribution shows the ratio between X and Y homologs. (DOCX 16 kb) Additional file 4: Figure S2. Combined padlock hybridization for *PCDH11X* and Y and Immunohistochemistry with Islet-1 antibodies in SC samples from human male embryos. a, b Padlock probe hybridization with probes for PCDH11X and PCDH11Y was combined with immunohistochemistry using islet-1 antibodies. Islet-1-positive cells are stained in yellow, PCDH11X signals are in sky blue and PCDH11Y in red. DAPI staining in dark blue allows visualization of nuclei. Part b is a detail enlargement of the complete SC section shown in a. (TIF 4288 kb) Additional file 5: Table S3. Description of the embryos and tissues used for each experiment. The table shows all embryonal sections and tissues used for RNA-seq, padlock probe and immunohistochemistry experiments. Embryos named E57, E49, E55 and E54 were used for RNA sequencing. The slides named E32_S62, E45_S71, E45_S72 and E45_S73 were included in the combined experiments of padlock probing and immunohistochemistry. Remaining embryonal sections were used for padlock probing experiments only. Experiments in which simultaneous detection of *PCDH11X*, *PCDH11Y*, *NLGN4X* and *NLGN4Y* was done are indicated by “PCDH11X/Y + NLGN4X/Y” in the column named “Gene”. (DOCX 19 kb) Additional file 6: Figure S3. Filtration of RNA sequencing data. The figure shows the reads mapped to the two last exons of NLGN4Y. Sequences mapping to more than one location in the genome are removed by filtration as described in the “Methods” section. After filtration, Y-specific sequences are not detected in females, indicating that the filtration process was effective in separating X- and Y-specific sequences. (TIF 1198 kb) Additional file 7: Figure S4. Alignment of transcripts for PCDH11X/Y and NLGN4X/Y. The colours represent percentage of identity in each region with dark blue corresponding to 100 % identity. The name of each transcript is given according to UCSC nomenclature with accession numbers in parenthesis. The limits between exon sequences are marked with red vertical lines. The exact position of each exon in the gene (hg 19) is given in Additional file 8: Table S4. The positions for padlock probes P1 to P5 are indicated at the bottom. In these positions, all X transcripts differ from all Y transcripts by one nucleotide. The exact positions (hg 19) of the nucleotide differences and the complete padlock probe sequences are shown in Additional file 9: Table S5. Alignments for transcripts of NLGN4X/Y. The positions of padlock probes N1 to N4 are indicated at the bottom. These probes are located in exons 3, 5 and 6. (TIF 869 kb) Additional file 8: Table S4. Percentage of sequence identity between exons of PCDH11X/Y and NLGNX/Y. Top. For each exon, the start and stop positions in the genome (hg 19) is given for NLGN4X and NLGN4Y, the size in base pairs, the percent of identity for X and Y exons, and the name of transcripts that contain the particular exon. Shaded in grey are those exons used for padlock probe design. Bottom. Same as above for exons contained in PCDH11X and PCDH11Y. The percentage of identity for X and Y exons was very high, ranging from 97 to 99 %. Notes: *This exon present in all transcripts except Y transcript uc004fte.2. **this exon also shares 87 % sequence identity to X position 5922232 to 5922326; ***repeated sequence with high sequence identity to sequences located in several chromosomes. ****high sequence identity also on chromosome 12. *****except Y transcript uc010nw.1. (DOCX 20 kb) Additional file 9: Table S5. Padlock probe sequences. The name and complete sequence for each probe is shown. In lowercase are the sequences included in the X and Y homologs for PCDH11, NLGN4 and *ACTB*. In uppercase are the sequences used for hybridization to detection oligos. Each PCDH11X/Y probe can be detected by two alternative detection oligos, allowing experiments in which only long transcripts or all transcripts are stained. In red colour are the base changes between all X and Y transcripts followed by the position of these bases in the genome. (DOCX 18 kb) Additional file 10: Table S6. Filters used for fluorescent imaging with the Zeiss Axio Imager.Z2 epi-fluorescence microscope. (DOCX 13 kb) Additional file 11: Figure S5. Cell segmentation using CellProfiler. The outlines of the cells defined by CellProfiler by nuclei segmentation. The outlines of the cells were defined at a 20-pixel distance from the outlines of the nuclei, which were identified by Global Otsu classification based on the DAPI nuclear staining. (TIF 2666 kb)
