# Supplementary material for: Spatial sexual dimorphism of X and Y homolog gene expression in the human central nervous system during early male development
Source: Biol Sex Differ. 2016 Jan 12;7:5. doi: 10.1186/s13293-015-0056-4 (PMC4710049; doi:10.1186/s13293-015-0056-4)
Supplement: Additional file 1: Table S1. — RNA sequencing results for all genes belonging to protocadherin and neuroligin families. The table shows expression values as base mean values for females and males, the position of each gene in the genome, fold change in positive values for male-biased genes and negative values for female-biased genes and p values adjusted for multiple testing as described in the “Methods” section. (DOCX 27 kb) [file 13293_2015_56_MOESM1_ESM.docx]

Supplementary Table 1. RNA sequencing results for all genes belonging to protocadherin and neuroligin families

| Gene | Base mean female | Base mean male | Chr | Start pos. | End pos. | Fold Change | P adj. | Bias |
| --- | --- | --- | --- | --- | --- | --- | --- | --- |
|  |  |  |  |  |  |  |  |  |
| NLGN4Y | 13,0 | 1191,3 | chrY | 16634488 | 16955848 | 91,77 | 1,9E-69 | 91,77 |
| NLGN4X | 3060,0 | 2037,8 | chrX | 5808083 | 6146706 | 0,67 | 0,334 | -1,50 |
| NLGN1 | 3230,9 | 4176,7 | chr3 | 173116244 | 174001116 | 1,29 | 1,000 | 1,29 |
| NLGN2 | 10059,9 | 10167,5 | chr17 | 7311502 | 7323179 | 1,01 | 1,000 | 1,01 |
| NLGN3 | 3735,7 | 3514,9 | chrX | 70364681 | 70391051 | 0,94 | 1,000 | -1,06 |
| NLGN4Y-AS1 | 0,0 | 0,0 | chrY | 16905522 | 16915913 | NA | NA | NA |
|  |  |  |  |  |  |  |  |  |
| PCDH11Y | 3,6 | 97,3 | chrY | 4868267 | 5610264 | 27,36 | 5,8E-07 | 27,36 |
| PCDHA9 | 685,9 | 381,6 | chr5 | 140227357 | 140391929 | 0,56 | 0,005 | -1,80 |
| PCDHA13 | 338,6 | 206,2 | chr5 | 140261854 | 140391929 | 0,61 | 0,057 | -1,64 |
| PCDHA10 | 317,8 | 246,5 | chr5 | 140235634 | 140391929 | 0,78 | 0,467 | -1,29 |
| PCDHGA4 | 595,9 | 622,7 | chr5 | 140734768 | 140892546 | 1,04 | 0,933 | 1,04 |
| PCDH1 | 4897,6 | 4275,6 | chr5 | 141232673 | 141257944 | 0,87 | 1,000 | -1,15 |
| PCDH10 | 4999,3 | 4053,4 | chr4 | 134070470 | 134112732 | 0,81 | 1,000 | -1,23 |
| PCDH11X | 263,6 | 269,3 | chrX | 91034260 | 91878228 | 1,02 | 1,000 | -0,98 |
| PCDH12 | 463,1 | 233,0 | chr5 | 141324530 | 141338627 | 0,50 | 1,000 | -1,99 |
| PCDH15 | 1323,0 | 1130,6 | chr10 | 55562533 | 56561051 | 0,85 | 1,000 | -1,17 |
| PCDH17 | 9005,3 | 7132,8 | chr13 | 58205789 | 58303065 | 0,79 | 1,000 | -1,26 |
| PCDH18 | 1756,9 | 1457,5 | chr4 | 138440074 | 138453629 | 0,83 | 1,000 | -1,21 |
| PCDH19 | 4988,2 | 3531,1 | chrX | 99546642 | 99665271 | 0,71 | 1,000 | -1,41 |
| PCDH20 | 205,9 | 152,2 | chr13 | 61983819 | 61989655 | 0,74 | 1,000 | -1,35 |
| PCDH7 | 6769,2 | 5774,5 | chr4 | 30722030 | 31148423 | 0,85 | 1,000 | -1,17 |
| PCDH8 | 881,2 | 764,6 | chr13 | 53418109 | 53422775 | 0,87 | 1,000 | -1,15 |
| PCDH9 | 9620,7 | 9724,4 | chr13 | 66876966 | 67804468 | 1,01 | 1,000 | 1,01 |
| PCDHA1 | 62,7 | 56,3 | chr5 | 140165876 | 140391929 | 0,90 | 1,000 | -1,11 |
| PCDHA11 | 368,8 | 359,4 | chr5 | 140247831 | 140391929 | 0,97 | 1,000 | -1,03 |
| PCDHA12 | 275,8 | 212,0 | chr5 | 140254931 | 140391929 | 0,77 | 1,000 | -1,30 |
| PCDHA2 | 117,3 | 117,8 | chr5 | 140174444 | 140391929 | 1,00 | 1,000 | 1,00 |
| PCDHA3 | 285,0 | 248,7 | chr5 | 140180783 | 140391929 | 0,87 | 1,000 | -1,15 |
| PCDHA4 | 238,0 | 215,6 | chr5 | 140186672 | 140391929 | 0,91 | 1,000 | -1,10 |
| PCDHA5 | 158,2 | 208,0 | chr5 | 140201361 | 140391929 | 1,31 | 1,000 | 1,31 |
| PCDHA6 | 235,7 | 184,8 | chr5 | 140207650 | 140391929 | 0,78 | 1,000 | -1,28 |
| PCDHA7 | 145,2 | 188,1 | chr5 | 140213969 | 140391929 | 1,30 | 1,000 | 1,30 |
| PCDHA8 | 132,4 | 113,3 | chr5 | 140220907 | 140391929 | 0,86 | 1,000 | -1,17 |
| PCDHAC1 | 665,4 | 593,9 | chr5 | 140306302 | 140391929 | 0,89 | 1,000 | -1,12 |
| PCDHAC2 | 538,7 | 516,8 | chr5 | 140345747 | 140391929 | 0,96 | 1,000 | -1,04 |
| PCDHB10 | 542,1 | 458,1 | chr5 | 140571952 | 140575213 | 0,85 | 1,000 | -1,18 |
| PCDHB11 | 374,5 | 354,1 | chr5 | 140579348 | 140582618 | 0,95 | 1,000 | -1,06 |
| PCDHB12 | 530,7 | 527,8 | chr5 | 140588291 | 140591698 | 0,99 | 1,000 | -1,01 |
| PCDHB13 | 352,1 | 307,4 | chr5 | 140593509 | 140596993 | 0,87 | 1,000 | -1,15 |
| PCDHB14 | 931,8 | 951,9 | chr5 | 140603078 | 140605860 | 1,02 | 1,000 | 1,02 |
| PCDHB15 | 840,1 | 689,6 | chr5 | 140625147 | 140627801 | 0,82 | 1,000 | -1,22 |
| PCDHB16 | 606,9 | 507,7 | chr5 | 140560980 | 140565796 | 0,84 | 1,000 | -1,20 |
| PCDHB17 | 117,5 | 86,8 | chr5 | 140535580 | 140537990 | 0,74 | 1,000 | -1,35 |
| PCDHB18 | 467,4 | 387,7 | chr5 | 140613938 | 140617101 | 0,83 | 1,000 | -1,21 |
| PCDHB19P | 638,3 | 542,3 | chr5 | 140619689 | 140624312 | 0,85 | 1,000 | -1,18 |
| PCDHB2 | 408,1 | 377,9 | chr5 | 140474237 | 140476964 | 0,93 | 1,000 | -1,08 |
| PCDHB3 | 488,0 | 436,0 | chr5 | 140480234 | 140483406 | 0,89 | 1,000 | -1,12 |
| PCDHB4 | 430,2 | 372,9 | chr5 | 140501581 | 140505201 | 0,87 | 1,000 | -1,15 |
| PCDHB5 | 419,0 | 359,0 | chr5 | 140514800 | 140517704 | 0,86 | 1,000 | -1,17 |
| PCDHB6 | 226,3 | 221,7 | chr5 | 140529839 | 140532868 | 0,98 | 1,000 | -1,02 |
| PCDHB7 | 408,2 | 397,0 | chr5 | 140552243 | 140555957 | 0,97 | 1,000 | -1,03 |
| PCDHB8 | 57,2 | 61,0 | chr5 | 140557371 | 140560081 | 1,06 | 1,000 | 1,06 |
| PCDHB9 | 421,3 | 333,6 | chr5 | 140566893 | 140571111 | 0,79 | 1,000 | -1,26 |
| PCDHGA1 | 79,1 | 106,2 | chr5 | 140710252 | 140892546 | 1,34 | 1,000 | 1,34 |
| PCDHGA10 | 713,9 | 593,3 | chr5 | 140792743 | 140892546 | 0,83 | 1,000 | -1,20 |
| PCDHGA11 | 1114,5 | 1027,3 | chr5 | 140800537 | 140892546 | 0,92 | 1,000 | -1,08 |
| PCDHGA12 | 519,1 | 457,9 | chr5 | 140810158 | 140892546 | 0,88 | 1,000 | -1,13 |
| PCDHGA2 | 380,8 | 340,5 | chr5 | 140718354 | 140892546 | 0,89 | 1,000 | -1,12 |
| PCDHGA3 | 258,9 | 191,6 | chr5 | 140723601 | 140892546 | 0,74 | 1,000 | -1,35 |
| PCDHGA5 | 586,2 | 586,2 | chr5 | 140743898 | 140892546 | 1,00 | 1,000 | -1,00 |
| PCDHGA6 | 671,2 | 641,8 | chr5 | 140753651 | 140892546 | 0,96 | 1,000 | -1,05 |
| PCDHGA7 | 624,7 | 537,5 | chr5 | 140762467 | 140892546 | 0,86 | 1,000 | -1,16 |
| PCDHGA8 | 426,9 | 438,0 | chr5 | 140771483 | 140892546 | 1,03 | 1,000 | 1,03 |
| PCDHGA9 | 873,8 | 912,7 | chr5 | 140782520 | 140892546 | 1,04 | 1,000 | 1,04 |
| PCDHGB1 | 303,2 | 342,6 | chr5 | 140729828 | 140892546 | 1,13 | 1,000 | 1,13 |
| PCDHGB2 | 549,5 | 535,5 | chr5 | 140739703 | 140892546 | 0,97 | 1,000 | -1,03 |
| PCDHGB3 | 527,7 | 564,8 | chr5 | 140749962 | 140892546 | 1,07 | 1,000 | 1,07 |
| PCDHGB4 | 449,5 | 576,8 | chr5 | 140767452 | 140892546 | 1,28 | 1,000 | 1,28 |
| PCDHGB5 | 524,1 | 521,7 | chr5 | 140777695 | 140892546 | 1,00 | 1,000 | -1,00 |
| PCDHGB6 | 1250,4 | 1274,4 | chr5 | 140787770 | 140892546 | 1,02 | 1,000 | 1,02 |
| PCDHGB7 | 1463,6 | 1356,5 | chr5 | 140797214 | 140892548 | 0,93 | 1,000 | -1,08 |
| PCDHGB8P | 491,9 | 465,7 | chr5 | 140805853 | 140807825 | 0,95 | 1,000 | -1,06 |
| PCDHGC3 | 1903,4 | 1895,8 | chr5 | 140855569 | 140892546 | 1,00 | 1,000 | -1,00 |
| PCDHGC4 | 3475,5 | 3039,8 | chr5 | 140864741 | 140892546 | 0,87 | 1,000 | -1,14 |
| PCDHGC5 | 535,6 | 507,5 | chr5 | 140868808 | 140892546 | 0,95 | 1,000 | -1,06 |
| PCDH9-AS2 | 0,0 | 0,0 | chr13 | 67399301 | 67489163 | NA | NA | NA |
| PCDH9-AS3 | 0,0 | 0,0 | chr13 | 67551521 | 67559908 | NA | NA | NA |
| PCDHB1 | 1,6 | 5,4 | chr5 | 140430979 | 140433512 | 3,26 | NA | 3,26 |
