# Supplementary figures and images for: Spatial sexual dimorphism of X and Y homolog gene expression in the human central nervous system during early male development
Source: Biol Sex Differ. 2016 Jan 12;7:5. doi: 10.1186/s13293-015-0056-4 (PMC4710049; doi:10.1186/s13293-015-0056-4)

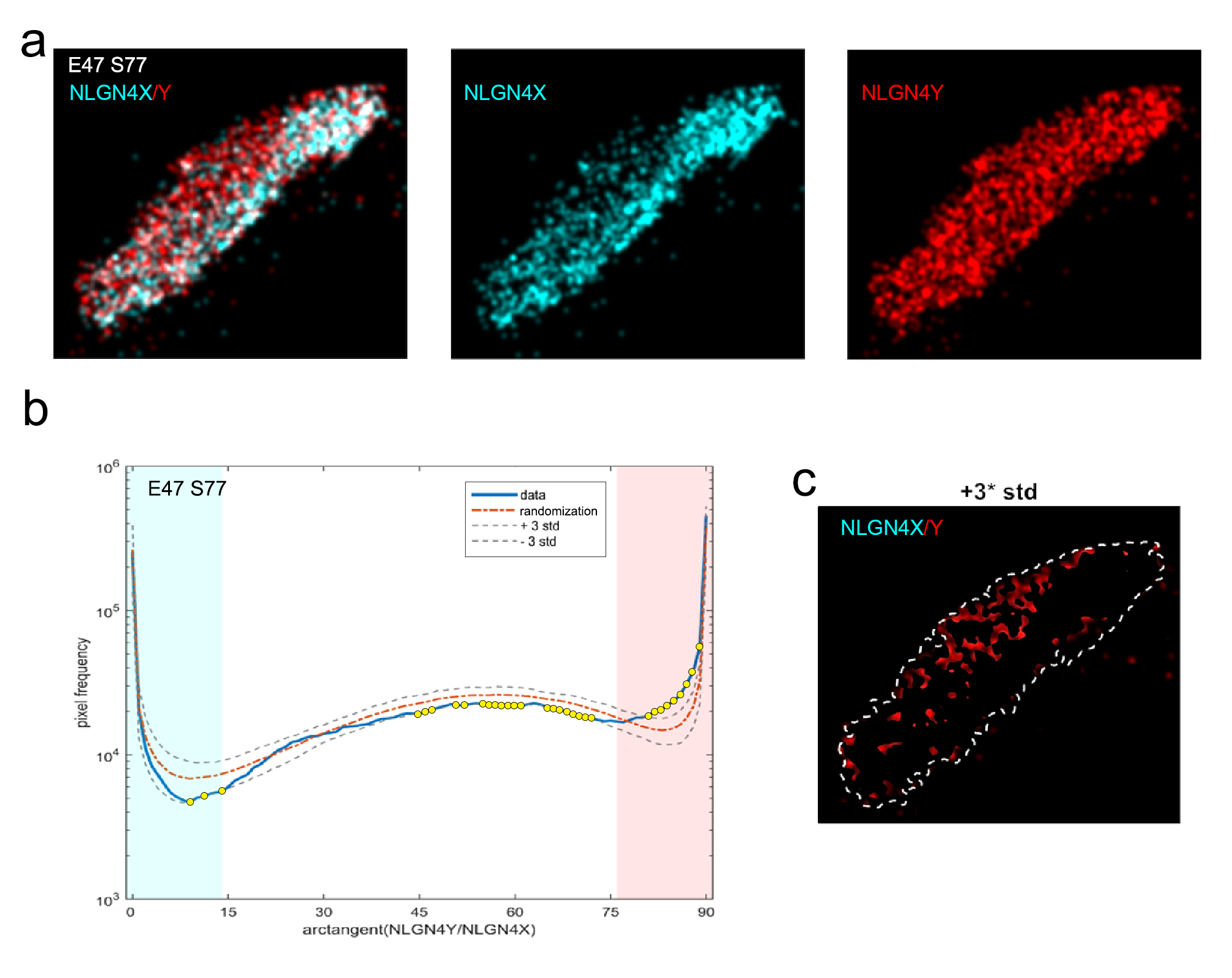

Supplement: Additional file 2: Figure S1. — Example of the objective analysis of spatial expression image data. Kernel density estimation (KDE) plots of PCDH11X/Y transcript signals detected in embryonic brain tissue are shown in a. At the top, the KDE plot of PCDH11X (cyan) has been merged with the one of PCDH11Y (red). From the separate KDE plots, two clear features can be distinguished; the Y homolog is more evenly expressed over the whole tissue while the X homolog shows higher expression in confined regions, namely along the lower edge and at the tip to the right. The observed spatial distribution of X and Y signals is compared to a random distribution in the histogram b, based on the relative contribution of X and Y signal intensity in each pixel in the KDE plot. The distribution of pixels in the KDE plot is shown as a blue line together with the average of 100 randomized data sets as a red line. Pixels deviating in number from what would be expected by chance (±3 SD from averaged random) are shown as a yellow dots on the blue line. The bar chart in b shows that we have a higher number of pixels classified as Y-dominant than we have X-dominant pixels, confirming our observations in the KDE plots in a that some regions express the Y homolog to a higher extent than the X homolog. Pixels in the observed signal distribution deviating more than 3 standard deviations from the random signal distribution, are plotted back onto the tissue in c and further confirms the patterns of X and Y homolog expression observed in a. The Y homolog shows dominant expression along the upper edge of the tissue section while the lower edge of the tissue section is dominated by mixed pixels (signal contribution from both the homologs). (TIF 623 kb) [file 13293_2015_56_MOESM2_ESM.tif]

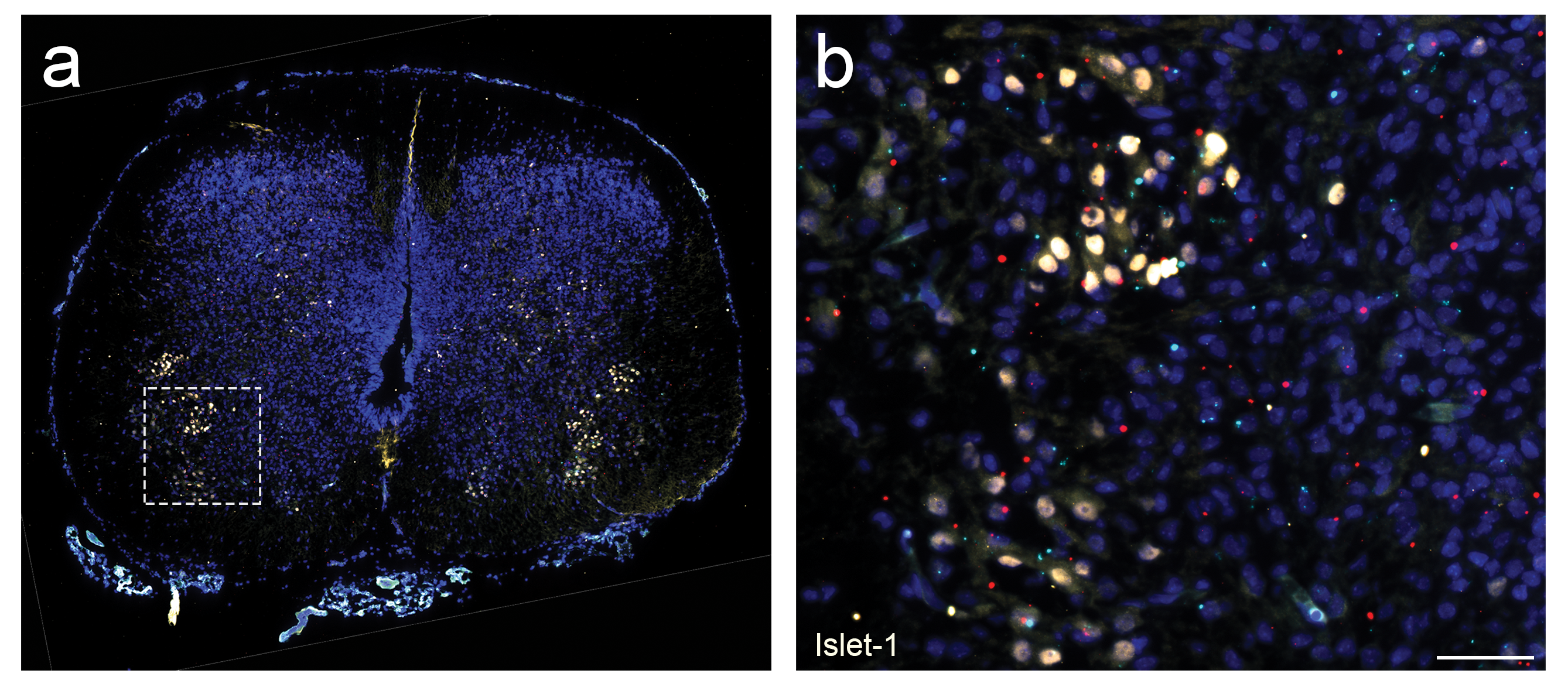

Supplement: Additional file 4: Figure S2. — Combined padlock hybridization for PCDH11X and Y and Immunohistochemistry with Islet-1 antibodies in SC samples from human male embryos. a, b Padlock probe hybridization with probes for PCDH11X and PCDH11Y was combined with immunohistochemistry using islet-1 antibodies. Islet-1-positive cells are stained in yellow, PCDH11X signals are in sky blue and PCDH11Y in red. DAPI staining in dark blue allows visualization of nuclei. Part b is a detail enlargement of the complete SC section shown in a. (TIF 4288 kb) [file 13293_2015_56_MOESM4_ESM.tif]

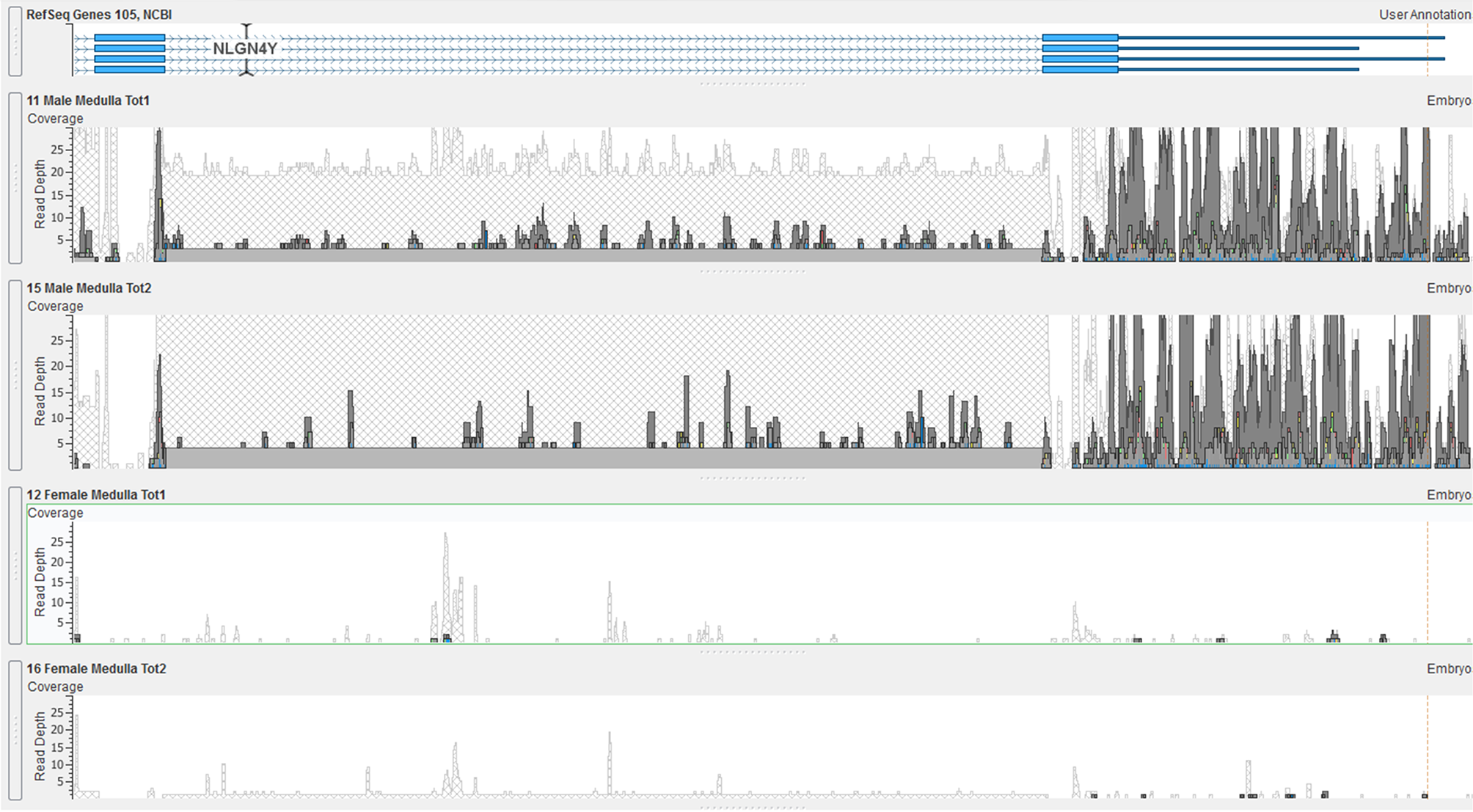

Supplement: Additional file 6: Figure S3. — Filtration of RNA sequencing data. The figure shows the reads mapped to the two last exons of NLGN4Y. Sequences mapping to more than one location in the genome are removed by filtration as described in the “Methods” section. After filtration, Y-specific sequences are not detected in females, indicating that the filtration process was effective in separating X- and Y-specific sequences. (TIF 1198 kb) [file 13293_2015_56_MOESM6_ESM.tif]

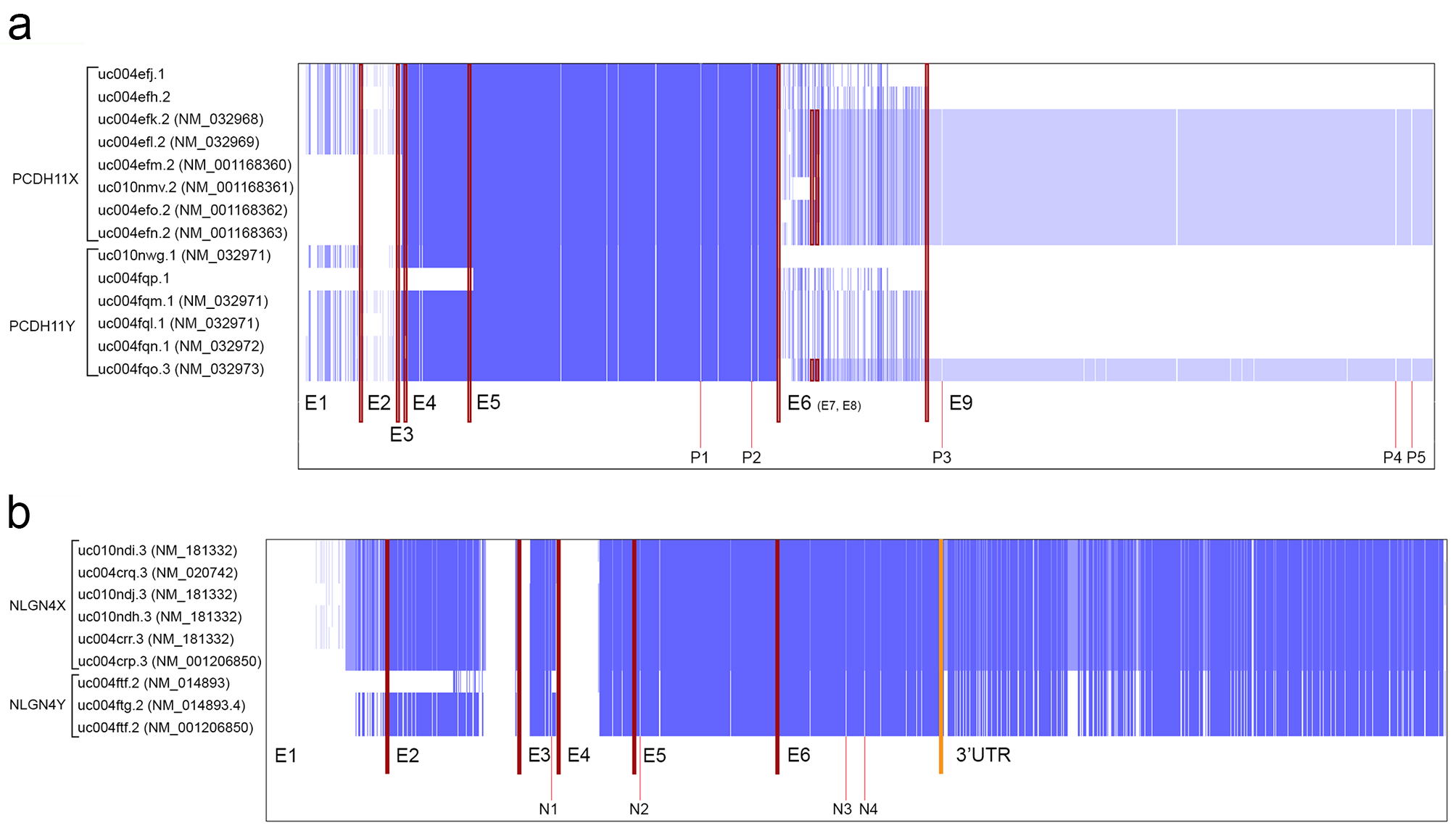

Supplement: Additional file 7: Figure S4. — Alignment of transcripts for PCDH11X/Y and NLGN4X/Y. The colours represent percentage of identity in each region with dark blue corresponding to 100 % identity. The name of each transcript is given according to UCSC nomenclature with accession numbers in parenthesis. The limits between exon sequences are marked with red vertical lines. The exact position of each exon in the gene (hg 19) is given in Additional file 8: Table S4. The positions for padlock probes P1 to P5 are indicated at the bottom. In these positions, all X transcripts differ from all Y transcripts by one nucleotide. The exact positions (hg 19) of the nucleotide differences and the complete padlock probe sequences are shown in Additional file 9: Table S5. Alignments for transcripts of NLGN4X/Y. The positions of padlock probes N1 to N4 are indicated at the bottom. These probes are located in exons 3, 5 and 6. (TIF 869 kb) [file 13293_2015_56_MOESM7_ESM.tif]

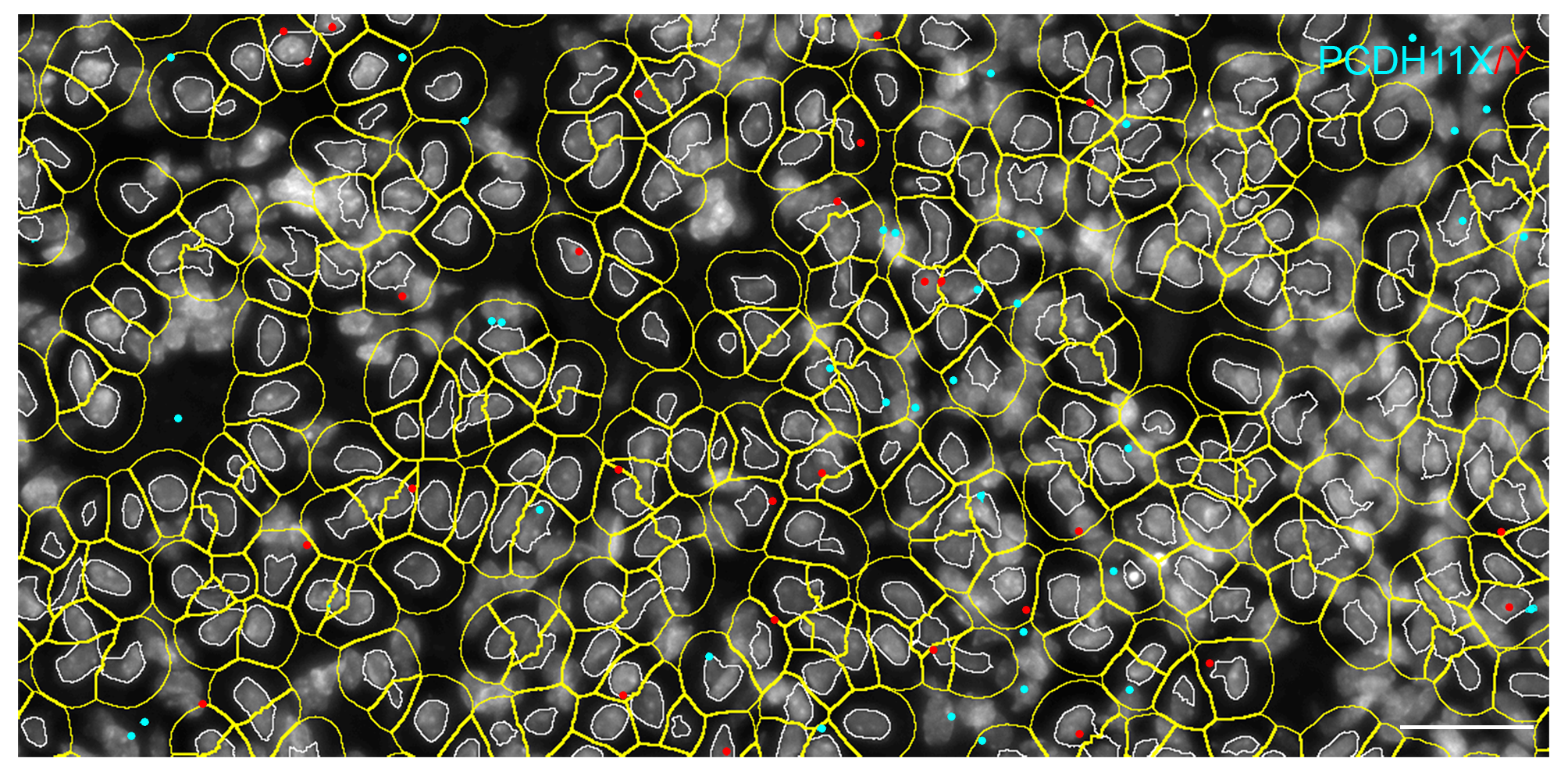

Supplement: Additional file 11: Figure S5. — Cell segmentation using CellProfiler. The outlines of the cells defined by CellProfiler by nuclei segmentation. The outlines of the cells were defined at a 20-pixel distance from the outlines of the nuclei, which were identified by Global Otsu classification based on the DAPI nuclear staining. (TIF 2666 kb) [file 13293_2015_56_MOESM11_ESM.tif]
