# Supplementary material for: Spatial sexual dimorphism of X and Y homolog gene expression in the human central nervous system during early male development
Source: Biol Sex Differ. 2016 Jan 12;7:5. doi: 10.1186/s13293-015-0056-4 (PMC4710049; doi:10.1186/s13293-015-0056-4)
Supplement: Additional file 3: Table S2. — Signal counts and statistical analysis for the ventral and dorsal part of the spinal cord. The table shows signal counts in dorsal vs. ventral SC sections acquired by using ImageJ software (Cell Counter plugin). Distributions for PCDH11X, PCDH11Y, NLGN4X and NLGN4Y are shown for slides obtained from seven female and four male embryos. Percentage distribution shows the ratio between X and Y homologs. (DOCX 16 kb) [file 13293_2015_56_MOESM3_ESM.docx]

| **Slide** | **Sex** | **Gene** | **Dorsal X** | **Ventral X** | **Dorsal Y** | **Ventral Y** | **Total X** | **Total Y** | **% Dorsal X** | **% Ventral X** | **% Dorsal Y** | **% Ventral Y** |
| --- | --- | --- | --- | --- | --- | --- | --- | --- | --- | --- | --- | --- |
| E31_S91 | Female | PCDH11 | 64 | 234 |  |  | 298 |  | 21% | 79% |  |  |
| E49_S82 | Female | PCDH11 | 130 | 214 |  |  | 344 |  | 38% | 62% |  |  |
| E49_S81 | Female | NLGN4 | 206 | 298 |  |  | 504 |  | 41% | 59% |  |  |
| E49_S82 | Female | NLGN4 | 172 | 239 |  |  | 411 |  | 42% | 58% |  |  |
| E27_S44 | Female | NLGN4 | 329 | 433 |  |  | 762 |  | 43% | 57% |  |  |
| E27_S48 | Female | NLGN4 | 287 | 412 |  |  | 699 |  | 41% | 59% |  |  |
| E31_S56 | Female | NLGN4 | 244 | 643 |  |  | 887 |  | 28% | 72% |  |  |
| E32_S84 | Male | PCDH11 | 185 | 296 | 142 | 347 | 481 | 489 | 38% | 62% | 29% | 71% |
| E32_S53 | Male | PCDH11 | 63 | 106 | 16 | 20 | 169 | 36 | 37% | 63% | 44% | 56% |
| E32_S29 | Male | NLGN4 | 237 | 360 | 366 | 432 | 597 | 798 | 40% | 60% | 46% | 54% |
| E32_S84 | Male | NLGN4 | 205 | 375 | 285 | 424 | 580 | 709 | 35% | 65% | 40% | 60% |

Supplementary Table 2. Signal counts and statistical analysis for the dorsal vs. ventral part of the spinal cord
