# Supplementary material for: Spatial sexual dimorphism of X and Y homolog gene expression in the human central nervous system during early male development
Source: Biol Sex Differ. 2016 Jan 12;7:5. doi: 10.1186/s13293-015-0056-4 (PMC4710049; doi:10.1186/s13293-015-0056-4)
Supplement: Additional file 5: Table S3. — Description of the embryos and tissues used for each experiment. The table shows all embryonal sections and tissues used for RNA-seq, padlock probe and immunohistochemistry experiments. Embryos named E57, E49, E55 and E54 were used for RNA sequencing. The slides named E32_S62, E45_S71, E45_S72 and E45_S73 were included in the combined experiments of padlock probing and immunohistochemistry. Remaining embryonal sections were used for padlock probing experiments only. Experiments in which simultaneous detection of PCDH11X, PCDH11Y, NLGN4X and NLGN4Y was done are indicated by “PCDH11X/Y + NLGN4X/Y” in the column named “Gene”. (DOCX 19 kb) [file 13293_2015_56_MOESM5_ESM.docx]

Supplementary Table 3

. Description of embryos and tissues used for each experiment

| **Sample ID** | **Sex** | **Week** | **Day** | **Tissue** | **Experiment** | **Gene** | **Antibody** |
| --- | --- | --- | --- | --- | --- | --- | --- |
| E31_S91 | Female | 9 | 3 | SC | Padlock probing | PCDH11XY |  |
| E27_S44 | Female | 11 | 0 | SC | Padlock probing | PCDH11XY |  |
| E31_S56 | Female | 9 | 3 | SC | Padlock probing | NLGN4XY |  |
| E27_S48 | Female | 11 | 0 | SC | Padlock probing | NLGN4XY |  |
| E49_S81 | Female | 11 | 2 | SC | Padlock probing | NLGN4XY |  |
| E49_S82 | Female | 11 | 2 | SC | Padlock probing | PCDH11XY + NLGN4XY |  |
| E57 | Female | 10 | 2 | MO | RNAseq |  |  |
| E34_S20 | Female | 11 | 0 | MO | Padlock probing | PCDH11XY |  |
| E33_S50 | Female | 10 | 6 | MO | Padlock probing | PCDH11XY |  |
| E33_S62 | Female | 10 | 6 | MO | Padlock probing | NLGN4XY |  |
| E34_S61 | Female | 11 | 0 | MO | Padlock probing | NLGN4XY |  |
| E33_S63 | Female | 10 | 6 | MO | Padlock probing | PCDH11XY + NLGN4XY |  |
| E34_S63 | Female | 11 | 0 | MO | Padlock probing | PCDH11XY + NLGN4XY |  |
| E49 | Female | 11 | 2 | Midbrain | RNAseq |  |  |
| E32_S53 | Male | 9 | 4 | SC | Padlock probing | PCDH11XY |  |
| E26_S65 | Male | 10 | 2 | SC | Padlock probing | NLGN4XY |  |
| E32_S29 | Male | 9 | 4 | SC | Padlock probing | NLGN4XY |  |
| E32_S84 | Male | 9 | 4 | SC | Padlock probing | PCDH11XY + NLGN4XY |  |
| E32_S62 | Male | 9 | 4 | SC | Padlock combined with Immuno | PCDH11XY | Islet 1 |
| E55 | Male | 10 | 2 | MO | RNAseq |  |  |
| E61_S49 | Male | 8 | 6 | MO | Padlock probing | PCDH11XY |  |
| E45_S75 | Male | 10 | 5 | MO | Padlock probing | PCDH11XY |  |
| E61_S39 | Male | 8 | 6 | MO | Padlock probing | NLGN4XY |  |
| E61_S89 | Male | 8 | 6 | MO | Padlock probing | NLGN4XY |  |
| E45_S48 | Male | 10 | 5 | MO | Padlock probing | NLGN4XY |  |
| E41_S58 | Male | 8 | 2 | MO | Padlock probing | PCDH11XY + NLGN4XY |  |
| E58_S82 | Male | 9 | 0 | MO | Padlock probing | PCDH11XY + NLGN4XY |  |
| E61_S72 | Male | 8 | 6 | MO | Padlock probing | PCDH11XY + NLGN4XY |  |
| E45_S50 | Male | 10 | 5 | MO | Padlock probing | PCDH11XY + NLGN4XY |  |
| E45_S71 | Male | 10 | 5 | MO | Padlock combined with Immuno | PCDH11XY | NeuN, Olig2 |
| E45_S72 | Male | 10 | 5 | MO | Padlock combined with Immuno | NLGN4XY | NeuN, Olig2 |
| E45_S73 | Male | 10 | 5 | MO | Padlock combined with Immuno | PCDH11XY, NLGN4XY | Sox10 |
| E54 | Male | 11 | 9 | Midbrain | RNAseq |  |  |
| E45_S330 | Male | 10 | 5 | Midbrain | Padlock probing | PCDH11XY |  |
| E47_S71 | Male | 11 | 6 | Midbrain | Padlock probing | PCDH11XY |  |
| E45_S329 | Male | 10 | 5 | Midbrain | Padlock probing | NLGN4XY |  |
| E47_S77 | Male | 11 | 6 | Midbrain | Padlock probing | NLGN4XY |  |
| E45_S329 | Male | 10 | 5 | Midbrain | Padlock probing | PCDH11XY + NLGN4XY |  |
| E47_S72 | Male | 11 | 6 | Midbrain | Padlock probing | PCDH11XY + NLGN4XY |  |
