# Supplementary material for: Spatial sexual dimorphism of X and Y homolog gene expression in the human central nervous system during early male development
Source: Biol Sex Differ. 2016 Jan 12;7:5. doi: 10.1186/s13293-015-0056-4 (PMC4710049; doi:10.1186/s13293-015-0056-4)
Supplement: Additional file 8: Table S4. — Percentage of sequence identity between exons of PCDH11X/Y and NLGNX/Y. Top. For each exon, the start and stop positions in the genome (hg 19) is given for NLGN4X and NLGN4Y, the size in base pairs, the percent of identity for X and Y exons, and the name of transcripts that contain the particular exon. Shaded in grey are those exons used for padlock probe design. Bottom. Same as above for exons contained in PCDH11X and PCDH11Y. The percentage of identity for X and Y exons was very high, ranging from 97 to 99 %. Notes: *This exon present in all transcripts except Y transcript uc004fte.2. **this exon also shares 87 % sequence identity to X position 5922232 to 5922326; ***repeated sequence with high sequence identity to sequences located in several chromosomes. ****high sequence identity also on chromosome 12. *****except Y transcript uc010nw.1. (DOCX 20 kb) [file 13293_2015_56_MOESM8_ESM.docx]

|  |  | | NLGN4Y | |  |  | NLGN4X |  |  | % | Transcript containing exon | |
| --- | --- | --- | --- | --- | --- | --- | --- | --- | --- | --- | --- | --- |
|  | Name (Fig. 1) | | Y Start position | | Y Stop position | size (bp) | X Start position | X Stop position | size (bp) | Identity Y/X | Y | X |
| 1 | E1 | | 16634488 | | 16634821 | 333 | 6146582 | 6146916 | 334 | 90 | uc004fte.2 | uc010ndj.3 |
| 2 |  | | 16635385 | | 16635452 | 67 | 6146054 | 6146123 | 69 | 89 | uc004fte.2 | None |
| 3 |  | | 16635626 | | 16635778 | 152 | 6145731 | 6145877 | 146 | 87 | uc004ftg.2 | None |
| 4 |  | | 16636454 | | 16636816 | 362 | 6144676 | 6145042 | 366 | 84 | uc004fth.2 | None |
| 5 | E2 | | 16733889 | | 16734471 | 582 | 6069036 | 6069601 | 565 | 94 | All * | All |
| 6 |  | | 16831339 | | 16831398 | 59 |  |  |  |  | uc004fte.2 | None |
| 7 | E3 | | 16834997 | | 16835149 | 152 | 5947321 | 5947473 | 152 | 93 | All | All |
| 8 |  | | 16845332 | | 16845429 | 97 | 5920532 | 5920626 | 94 | 89 | uc004fti.4 | None** |
| 9 |  | | 16860499 | | 16860609 | 110 | 5909453 | 5909560 | 107 | 97 | uc004ftf.2 | None |
| 10 |  | | 16863561 | | 16863682 | 121 |  |  |  |  | uc004ftf.2 | *** |
| 11 | E4 | | 16936068 | | 16936253 | 185 | 5827095 | 5827280 | 185 | 98 | All | All |
| 12 | E5 | | 16941610 | | 16942399 | 789 | 5821118 | 5821907 | 789 | 98 | All | All |
| 13 | E6 | | 16952293 | | 16955848 | 3555 | 5808070 | 5811707 | 3637 | 89 | All | All |
|  |  | |  | |  |  |  |  |  |  |  |  |
|  | **PCDH11X/Y EXONS** | | | | |  |  |  |  |  |  |  |
|  |  | PCDH11Y | |  | |  | PCDH11X |  |  |  |  |  |
| 1 | E1 | 4868267 | | 4868646 | | 379 | 91034304 | 91034683 | 379 | 99 | All short | uc004efh.2 |
| 2 | E2 | 4899947 | | 4900052 | | 105 | 91065472 | 91065577 | 105 | 97 | uc004fql.1 | **** |
| 3 | E3 | 4900711 | | 4900769 | | 58 | 91066236 | 91066294 | 58 | 97 | All short | uc004efh.2 |
| 4 |  | 4924131 | | 4925500 | | 1369 | 91089661 | 91091043 | 1382 | 98 | uc004fqo.3 |  |
| 5 | E4 | 4924930 | | 4925500 | | 570 | 91090460 | 91091043 | 583 | 97 | All short | All |
| 6 | E5 | 4966256 | | 4968748 | | 2492 | 91131780 | 91134272 | 2492 | 99 | All | All |
| 7 | E6 | 4972385 | | 4973485 | | 1100 | 91137906 | 91139006 | 1100 | 99 | All short***** | uc004efh.2 |
| 8 | E7 | 5369098 | | 5369296 | | 198 | 91642734 | 91642932 | 198 | 99 | uc004fqo.3 | All long |
| 9 | E8 | 5449816 | | 5449839 | | 23 |  |  |  |  | uc004fqo.3 | *** |
| 10 | E9 | 5605313 | | 5610264 | | 4951 | 91873269 | 91878228 | 4959 | 98 | uc004fqo.3 | All long |

Supplementary Table 4. Percentage of sequence identity between exons of PCDH11X/Y and NLGN4X/Y
