# Supplementary material for: Spatial sexual dimorphism of X and Y homolog gene expression in the human central nervous system during early male development
Source: Biol Sex Differ. 2016 Jan 12;7:5. doi: 10.1186/s13293-015-0056-4 (PMC4710049; doi:10.1186/s13293-015-0056-4)
Supplement: Additional file 9: Table S5. — Padlock probe sequences. The name and complete sequence for each probe is shown. In lowercase are the sequences included in the X and Y homologs for PCDH11, NLGN4 and ACTB. In uppercase are the sequences used for hybridization to detection oligos. Each PCDH11X/Y probe can be detected by two alternative detection oligos, allowing experiments in which only long transcripts or all transcripts are stained. In red colour are the base changes between all X and Y transcripts followed by the position of these bases in the genome. (DOCX 18 kb) [file 13293_2015_56_MOESM9_ESM.docx]

|  |  | Position | Exon |  |
| --- | --- | --- | --- | --- |
| Name | Sequence | (hg19) | (Fig1) | Detection Oligo |
| PdPX_1 | tgaagcaccagtgaccccaaTCTTCTACTGGACCTTAATCGTGTGCGCCTCAATGCACATGTTTGGCTCCgaactggtgcgcaaaagcaC | 91133608 | E5 | B2_DO_33+, Lin33 |
| PdPY_1 | tgaagcaccagtgaccccaaTCTTCTAAGTCGGAAGTACTACTCTCTCCTCAATGCTGCTGCTGTACTACgaactggtgcgcaaaagcaT | 4968084 | E5 | Allel2, Lin16 |
| PdPX_2 | gacagccctgatttggcccgTCTTCTACTGGACCTTAATCGTGTGCGCCTCAATGCACATGTTTGGCTCCcacctactactttcaagccC | 91134029 | E5 | B2_DO_33+, Lin33 |
| PdPY_2 | gacagccctgatttggcccgTCTTCTAAGTCGGAAGTACTACTCTCTCCTCAATGCTGCTGCTGTACTACcacctactactttcaagccT | 4968505 | E5 | Allel2, Lin16 |
| PdPX_3 | actatttctgatgtacagtaTCTTCTATGCGTCTATTTAGTGGAGCCCCTCAATGCACATGTTTGGCTCCggcttatcatgacagagcgC | 91874201 | E9 | B2_DO_27+, Lin33 |
| PdPY_3 | actatttctgatgtacagtaTCTTCTACCGAGATGTACCGCTATCGTCCTCAATGCTGCTGCTGTACTACggcttatcatgacagagcgT | 5606245 | E9 | Allel1, Lin16 |
| PdPX_4 | tgtaagtgatactttttaaaTCTTCTATGCGTCTATTTAGTGGAGCCCCTCAATGCACATGTTTGGCTCCaatggcaattgtttagtgaT | 91877912 | E9 | B2_DO_27+, Lin33 |
| PdPY_4 | tgtaagtgatactttttaaaTCTTCTACCGAGATGTACCGCTATCGTCCTCAATGCTGCTGCTGTACTACaatggcaattgtttagtgaC | 5609948 | E9 | Allel1, Lin16 |
| PdPX_5 | catgctttggaagtaagaagTCTTCTATGCGTCTATTTAGTGGAGCCCCTCAATGCACATGTTTGGCTCCtatcccaaagcagttccaaC | 91878080 | E9 | B2_DO_27+, Lin33 |
| PdPY_5 | catgctttggaagtaagaagTCTTCTACCGAGATGTACCGCTATCGTCCTCAATGCTGCTGCTGTACTACtatcccaaagcagttccaaG | 5610116 | E9 | Allel1, Lin16 |
| pLNA_P1 | ggatacatcagctatctcagtattt |  |  |  |
| pLNA_P2 | gtggagaggcagatttgtagtgtcg |  |  |  |
| pLNA_P3 | atgatgataaaaacaacaaaaaata |  |  |  |
| pLNA_P4 | agtataaatttcacacagtttactc |  |  |  |
| pLNA_P5 | ccttggccatacaatagtcaacctt |  |  |  |
| PdNX_1 | aacgtcatcgtgatcaccatTCTTCTATCTTCTTTCGTGCGCCTGGTAGCAAATACCTTTCCTTTTACGAgcattttggcaagctacggA | 5947364 | E3 | B2_DO_30 |
| PdNY_1 | aacgtcatcgttatcaccatTCTTCTATCTTCTTTGCGCCACTGATACTAGATTGCCTTTCCTTTTACGAgcattttggccagctatggG | 16835106 | E3 | B2_DO_32- |
| PdNX_2 | gccctgtccagctgggcagtTCTTCTATCTTCTTTCGTGCGCCTGGTAGCAAATACCTTTCCTTTTACGAccatcattcagagcggcacC | 5821873 | E5 | B2_DO_30 |
| PdNY_2 | gccctgtccagctgggcagtTCTTCTATCTTCTTTGCGCCACTGATACTAGATTGCCTTTCCTTTTACGAccatcattcagagcggcacT | 16941644 | E5 | B2_DO_32- |
| PdNX_3 | gaggacacaactgtcctcatTCTTCTATCTTCTTTCGTGCGCCTGGTAGCAAATACCTTTCCTTTTACGAaccctcacaaaacagggccT | 5811335 | E6 | B2_DO_30 |
| PdNY_3 | gaggacacaactgtcctcatTCTTCTATCTTCTTTGCGCCACTGATACTAGATTGCCTTTCCTTTTACGAaccctcacaaaacagggccC | 16952665 | E6 | B2_DO_32- |
| PdNX_4 | tttgcggcgctgtactacaaTCTTCTATCTTCTTTCGTGCGCCTGGTAGCAAATACCTTTCCTTTTACGAtcttcctcaacatcttagcT | 5811233 | E6 | B2_DO_30 |
| PdNY_4 | tttgcggcgctgtactacaaTCTTCTATCTTCTTTGCGCCACTGATACTAGATTGCCTTTCCTTTTACGAtcttcctcaacatcttagcC | 16952767 | E6 | B2_DO_32- |
| pLNA_N_1 | tagtattcccagacggtagttaatg |  |  |  |
| pLNA_N_2 | gtacttggccggctggtagttcact |  |  |  |
| pLNA_N_3 | gaataatctcgtttggtttcaatga |  |  |  |
| pLNA_N_4 | ctcatggcgcctcttgtcctttttg |  |  |  |
|  |  |  |  |  |
|  | Sequence detection oligos: |  |  |  |
| B2_DO_27+ | TGCGTCTATTTAGTGGAGCC |  |  |  |
| B2_DO_33+ | CTGGACCTTAATCGTGTGCG |  |  |  |
| Lin16 | CCTCAATGCTGCTGCTGTACTAC |  |  |  |
| Lin33 | CCTCAATGCACATGTTTGGCTCC |  |  |  |
| Allel1 | CCGAGATGTACCGCTATCGT |  |  |  |
| Allel2 | AGTCGGAAGTACTACTCTCT |  |  |  |
| B2_DO_32- | GCGCCACTGATACTAGATTG |  |  |  |
| B2_DO_30 | CGTGCGCCTGGTAGCAAATA |  |  |  |
| Lin16 | CCTCAATGCTGCTGCTGTACTAC |  |  |  |
| Lin33 | CCTCAATGCACATGTTTGGCTCC |  |  |  |

Supplementary Table 5. Padlock probe and detection oligo sequences
