# Supplementary material for: Spatial sexual dimorphism of X and Y homolog gene expression in the human central nervous system during early male development
Source: Biol Sex Differ. 2016 Jan 12;7:5. doi: 10.1186/s13293-015-0056-4 (PMC4710049; doi:10.1186/s13293-015-0056-4)
Supplement: Additional file 10: Table S6. — Filters used for fluorescent imaging with the Zeiss Axio Imager.Z2 epi-fluorescence microscope. (DOCX 13 kb) [file 13293_2015_56_MOESM10_ESM.docx]

**Supplementary Table 6.** Filters used for fluorescent imaging with the Zeiss Axio Imager .Z2 epi-fluorescence microscope.

| **Filter name** | **Fluorophore** |
| --- | --- |
| 49DAPI, Zeiss | Hoechst |
| 38HE, Zeiss | FAM/FITC |
| Cy3 (SP102v2_Zeiss Axio_2-8 Cy3 v2, Chroma) | Cy3 |
| Cy3.5 (SP103v2_Zeiss Axio_2-8 Cy3.5 v2, Chroma) | TexasRed |
| Cy5 (SP104v2_Zeiss Axio_2-8_Mounted Cy5 v2, Chroma) | Cy5 |
| Cy7 (49007_Zeiss Axio_2-8_Mounted ET- CY7, Chroma) | AlexaFluor750 |
